# Activation of the impaired NAMPT/SIRT7/SOD2 axis restores alveolar progenitor cell renewal in idiopathic pulmonary fibrosis

**DOI:** 10.1172/JCI198031

**Published:** 2026-05-07

**Authors:** Xuexi Zhang, Xue Liu, Yujie Qiao, Anas Rabata, Ningshan Liu, Changfu Yao, Tanyalak Parimon, Danica Chen, Cory Hogaboam, Peter Chen, Barry Stripp, Stephen J. Gardell, Dianhua Jiang, Paul W. Noble, Jiurong Liang

**Affiliations:** 1 Department of Medicine and Women’s Guild Lung Institute, Cedars-Sinai Medical Center, Los Angeles, California, USA.; 2 Department of Nutritional Science and Toxicology, University of California, Berkeley, California, USA.; 3 Department of Biomedical Sciences, Cedars-Sinai Medical Center, Los Angeles, California, USA.; 4 Advent Health Translational Research Institute, Orlando, Florida, USA.; 5 Department of Medicine, David Geffen School of Medicine at UCLA, Los Angeles, California, USA.

**Keywords:** Cell biology, Metabolism, Pulmonology, Adult stem cells, Fibrosis, Mitochondria

## Abstract

Alveolar type 2 (AT2) progenitor cell exhaustion and impaired regenerative capacity are key pathogenic hallmarks in idiopathic pulmonary fibrosis (IPF). Nicotinamide adenine dinucleotide (NAD^+^) functions as a central regulator of cellular energy metabolism. We have previously reported that downregulation of NAD^+^-dependent sirtuin signaling contributes to the impaired progenitor cell function of IPF AT2 cells. In this study, we found that a key NAD^+^ biosynthesis enzyme, nicotinamide phosphoribosyltransferase (NAMPT), was significantly downregulated in IPF AT2 cells. NAMPT deficiency impaired AT2 renewal and enhanced lung fibrosis through downregulation of SIRT7 and SOD2, which resulted in increased oxidative stress, mitochondrial dysfunction, accumulated aberrant transitional cells, and impaired differentiation from AT2 to alveolar type 1 (AT1) cells. A mouse model with AT2-specific deletion of Nampt showed severely impaired AT2 renewal capacity and increased susceptibility to bleomycin lung injury. Activation of NAMPT by small-molecule activators promoted IPF AT2 renewal and reversed lung fibrosis in WT mice. NAMPT activation is a potentially promising therapeutic strategy for restoring AT2 progenitor cell function and halting or reversing progressive pulmonary fibrosis.

## Abstract 2

Boosting a key NAD^+^ enzyme, NAMPT, restores lung cell regeneration and reduces fibrosis, offering a potential treatment for idiopathic pulmonary fibrosis.

## Graphical abstract

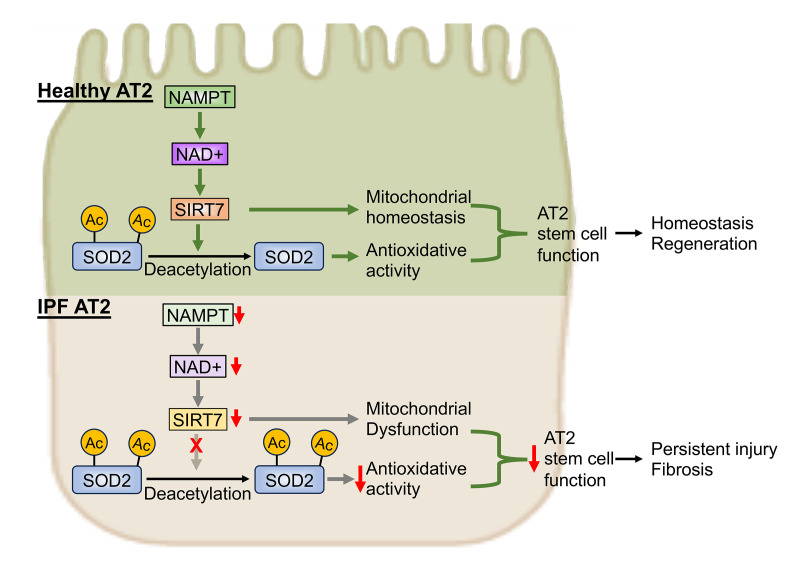

## Introduction

Idiopathic pulmonary fibrosis (IPF) is a fatal interstitial lung disease with limited treatment options (1–3). The median survival of patients with IPF from diagnosis is 2–4 years (3). Despite FDA-approved treatments with limited therapeutic effect in restoring lung functions (4, 5), IPF remains the leading indication of lung transplantation. Loss of epithelial integrity due to repeated injury and inadequate alveolar repair is a seminal causal event in IPF pathogenesis (2, 6–12). Type 2 alveolar (AT2) epithelial cells function as progenitor cells to maintain alveolar integrity during homeostasis and in response to injury (13–15). The AT2 cell population in IPF lungs is decreased, and the remaining AT2 cells in the diseased lungs have impaired renewal capacity and fail to generate type 1 alveolar (AT1) epithelial cells that are essential for gas exchange (6, 8, 16). However, the molecular mechanisms that control AT2 progenitor cell renewal and unremitting lung fibrosis remain poorly understood.

Nicotinamide adenine dinucleotide (NAD^+^) and its reduced derivative, NADH, are central regulators of cell energy metabolism (17, 18). NAD^+^ levels decline with aging in various tissues including heart, lung, liver, muscle, and skin (19, 20). Published studies showed that NAD^+^ levels are decreased in IPF lungs (21, 22), and NAD^+^ deficiency is associated with lung fibrosis (21–23). However, the specific alterations in NAD^+^ metabolic pathways and underlying mechanisms within AT2 progenitor cells in fibrotic lungs remain incompletely explored.

NAD^+^ is synthesized via the de novo, Preiss-Handler, and salvage pathways, which use tryptophan, nicotinic acid, and nicotinamide (NAM), respectively, as the precursor for the pyridine ring moiety (19, 24, 25). The salvage pathway is the major contributor to NAD^+^ synthesis in a wide variety of tissues (24, 26), including the lungs. Nicotinamide phosphoribosyltransferase (NAMPT), as the rate-determining enzyme in the NAD^+^ salvage pathway, plays a crucial role in producing nicotinamide mononucleotide (NMN), which is subsequently converted to NAD^+^ by NMN adenylyltransferase (NMNAT) (19, 24). NAMPT regulates stem cell functions, mitochondrial bioenergetics, as well as cell apoptosis and senescence in neurodegenerative diseases, endothelial cells, and human induced pluripotent stem cells (iPSCs) (27–31). NAMPT-mediated NAD^+^ biosynthesis protects against CCl_4_-induced liver fibrosis (32). Previous efforts to restore NAD^+^ have focused on using precursors such as NMN, NAM, and NR (nicotinamide riboside) (25), and greater attention is now shifting toward an emerging class of small molecules known as NAMPT activators, which directly bind to NAMPT to boost NMN production and enhance NAD^+^ biosynthesis (28, 33). The development of NAMPT activators as targeted NAD^+^ boosters is especially important, as they provide a more effective and precise way to increase NAD^+^ levels, with substantial therapeutic potential for a range of aging-associated diseases (28, 33).

NAD^+^ is a cosubstrate for sirtuins (SIRT1–7) (19, 24), a family of NAD^+^-dependent deacylases that have been implicated in promoting stem cell function and cell rejuvenation (18, 34, 35). We recently reported that downregulation of NAD^+^-dependent sirtuin signaling contributes to the impaired progenitor cell function of IPF AT2 cells and that NAD^+^ precursors promote AT2 renewal (8). Among sirtuins, SIRT7 regulates cellular energy consumption (36) and mitochondrial homeostasis (37, 38). SIRT7 expression is reduced in aging (39–41), whereas its upregulation improves regeneration of aged hematopoietic stem cells (39), and protects against cellular senescence in mammalian cell lines (42) and mesenchymal stem cells (43). Hepatic SIRT7 suppresses ER stress and prevents fatty liver disease (44). The NAD^+^ precursor NMN promotes intestinal stem cell proliferation, reduces cellular ROS levels, and protects against radiation injury by enhancing SIRT6 and SIRT7 activities (45). Given the widely recognized roles of NAD^+^ and SIRT7 in stem cell function and stress resilience, their intertwined roles in regulating AT2 progenitor cell homeostasis in IPF and lung fibrosis remain poorly understood.

NAD^+^ is a ubiquitous enzyme cofactor that is obligatory for cellular redox reactions, and its decline contributes to oxidative stress and mitochondrial dysfunction in aging and aging-associated diseases (46, 47). Epithelial cells in IPF lungs show increased oxidative stress (48–52) and diminished expression of oxidative response proteins (53–55), leading to senescence and apoptosis. Among key protective factors, SOD2 plays a crucial role in shielding alveolar epithelial cells from lung injury (56, 57), while SIRT7 maintains mitochondrial homeostasis, enhances cell proliferation, and suppresses both apoptosis and oxidative stress (58–60). However, the possible interaction between SIRT7 and SOD2 to preserve AT2 progenitor cell homeostasis remains unexplored.

In this study, we identified downregulation of NAMPT, the rate-limiting enzyme in NAD^+^ biosynthesis, in AT2 cells from IPF explants. Functional analyses demonstrated that enhancing NAMPT activity, either genetically or pharmacologically, promoted, whereas NAMPT inhibition impaired, AT2 progenitor cell renewal in 3D organoid cultures in vitro and mouse models in vivo. Mechanistically, NAMPT regulates AT2 renewal through SIRT7 and SOD2. NAMPT deficiency in IPF AT2 cells leads to downregulation of SIRT7 and SOD2, increasing cell vulnerability to oxidative stress and mitochondrial dysfunction, which ultimately elicits impaired AT2 cell renewal and severe lung fibrosis. Notably, NAMPT activators increased AT2 recovery and attenuated bleomycin-induced lung fibrosis in both young and aged mice. To our knowledge, this is the first study showing that the NAMPT/SIRT7/SOD2 axis plays a critical role in regulating AT2 renewal and highlights potential therapeutic approaches, such as NAMPT activators, for IPF intervention.

## Results

### Decreased expression of a NAD^+^ biosynthesis enzyme, NAMPT, in IPF AT2 cells.

**Figure 1:**
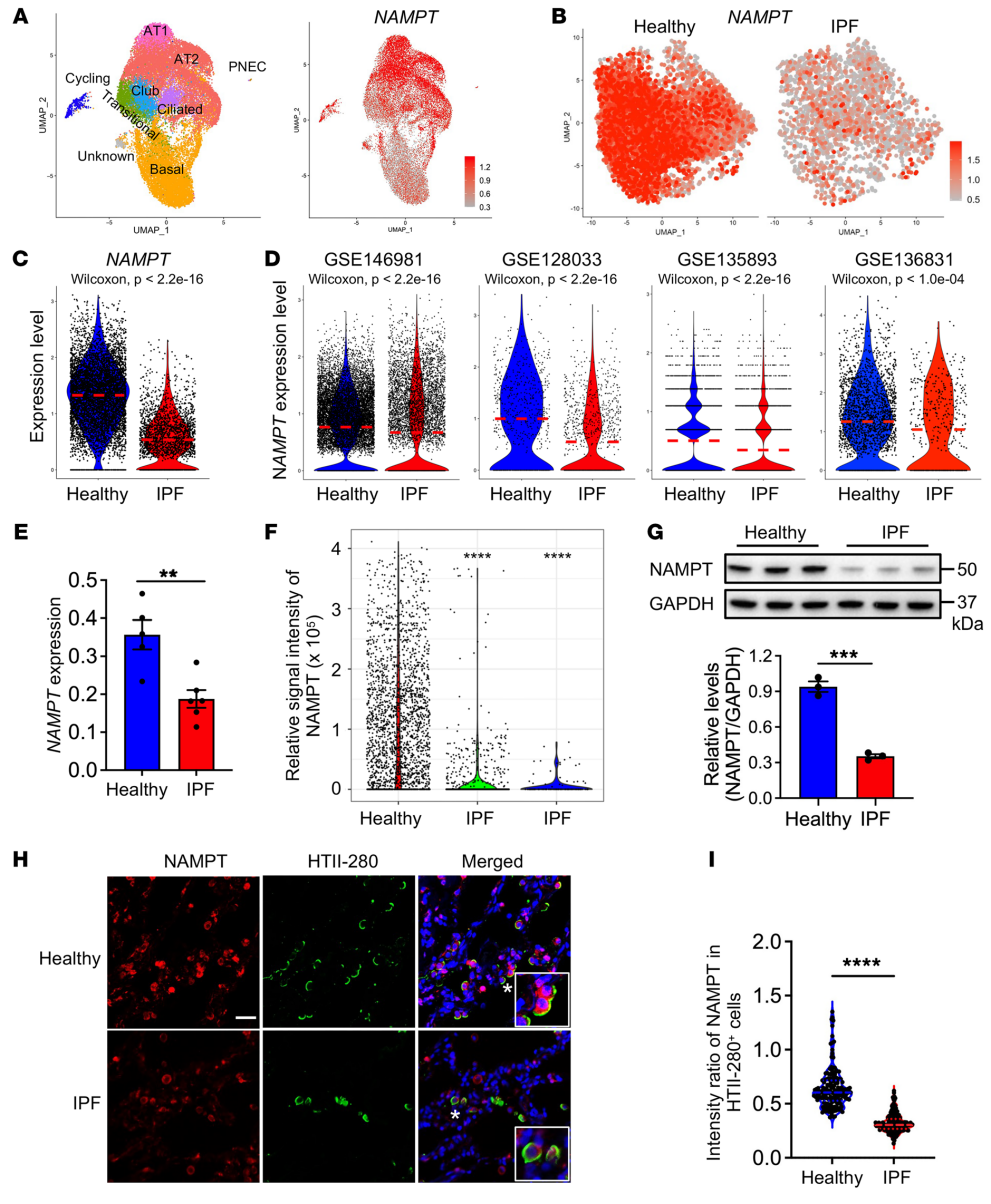
Decreased expression of the NAD^+^ biosynthesis enzyme NAMPT in IPF AT2 cells. (**A**) Uniform manifold approximation and projection (UMAP) visualization of epithelial cell types and *NAMPT* expression in healthy and IPF lung cells in the in-house scRNA-seq dataset. PNEC, pulmonary neuroendocrine cells. (**B**) UMAP visualization of *NAMPT* expression in AT2 cells from healthy and IPF lungs. (**C** and **D**) Violin plots showing *NAMPT* expression levels in AT2 cells from in-house (**C**) and recently published scRNA-seq datasets (**D**). (**E**) Real-time PCR analysis of *NAMPT* expression in freshly isolated AT2 cells from healthy and IPF lungs (*n* = 5–6/group; **P < 0.01, by unpaired, 2-tailed *S*tudent’s *t* test). (**F**) Single-cell Western blot analysis of NAMPT expression in freshly isolated AT2 cells from healthy and IPF lungs, with β-tubulin used as a loading control (****P < 0.0001, by 1-way ANOVA). (**G**) Western blot analysis and quantification of NAMPT expression in immortalized AT2 cells from healthy and IPF donors. GAPDH served as a loading control (*n* = 3/group; ***P < 0.001, by unpaired, 2-tailed *S*tudent’s *t* test). (**H**) Costaining of NAMPT (red) and HTII-280 (green, an AT2 marker) of healthy and IPF lung sections. The staining was performed with lung sections from 3 patients with IPF and 3 healthy donors. Representative cells (asterisks) are shown at higher magnification. Scale bars: 20 μm. Insets, original magnification, ×63. (**I**) Quantification of NAMPT staining (relative signal intensity) in individual AT2 cells (HTII-280^+^), shown as violin plots with median and quartiles (*n* = 50–60 cells/section, *n* = 3/group; ****P < 0.0001, by unpaired, 2-tailed *S*tudent’s *t* test). Data are shown as the mean ± SEM. All experiments were repeated at least 3 times.

NAD^+^ levels are decreased in IPF lung tissues (21, 22). The salvage pathway involving NAMPT is the major route for NAD^+^ biosynthesis in the lung (26). We recently published a single-cell RNA-seq (scRNA-seq) analysis using flow-enriched human lung epithelial cells from healthy and IPF lungs (8). Reanalysis with updated epithelial cell annotations and commonly used gene signatures refined the classification of epithelial subpopulations (Figure 1A and Supplemental Figure 1, A and B; supplemental material available online with this article; https://doi.org/10.1172/JCI198031DS1). Notably, NAMPT was found to be highly expressed in AT2 cells (Figure 1A), which was confirmed by integrative analysis of multiple published scRNA-seq datasets (61–64) (Supplemental Figure 1C). Interestingly, expression levels of NAMPT were dramatically lower in AT2 cells from IPF lungs compared with levels in healthy controls (Figure 1, B and C), which was also further evidenced in multiple recently published scRNA-seq datasets (61–64) (Figure 1D). Using freshly isolated AT2 cells from healthy and IPF lungs, we further showed decreased NAMPT expression at both mRNA levels by qPCR (Figure 1E) and protein levels by both single cell (Figure 1F) and conventional Western blotting (Figure 1G) in IPF AT2 cells. Immunofluorescence showed that NAMPT was colocalized with the human AT2 marker HTII-280 (Figure 1H), and fluorescence intensity of NAMPT staining in AT2 cells of IPF lungs was decreased compared with healthy controls (Figure 1, H and I).

We also compared NAMPT expression in fibroblasts from fibrotic lung tissues with integrated scRNA-seq data analysis (65). The expression levels of NAMPT were not significantly different between fibroblasts from healthy and IPF human lungs (Supplemental Figure 1D), or from bleomycin-induced fibrotic and control mouse lungs (Supplemental Figure 1E).

### NAMPT regulates AT2 progenitor cell regeneration.

**Figure 2:**
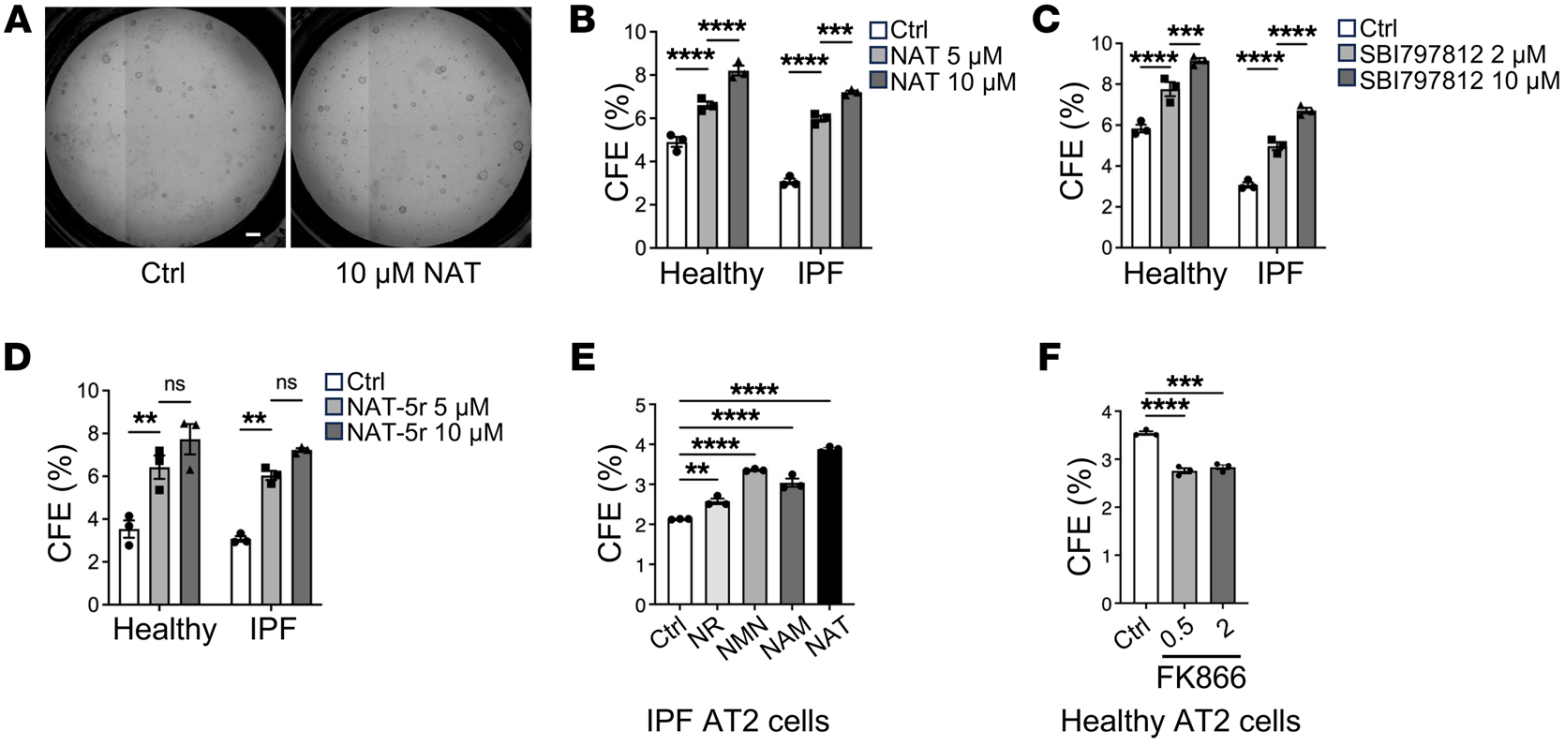
NAMPT regulates AT2 progenitor cell regeneration. (**A**) Representative images of human AT2 colonies cultured with NAT or DMSO control (Ctrl). Scale bar: 300 μm. (**B**–**D**) CFE in 3D organoid cultures of AT2 cells from healthy and IPF lungs treated with the NAMPT activators (**B**) NAT, (**C**) SBI797812, or (**D**) NAT-5r (*n* = 3/group; **P < 0.01, ***P < 0.001, ****P < 0.0001, by 2-way ANOVA). (**E**) CFE in 3D organoid cultures of AT2 cells from IPF lungs treated with the NAMPT precursors NR, NMN, or NAM, as well as the NAMPT activator NAT (*n* = 3/group; **P < 0.01, ****P < 0.0001, by 1-way ANOVA). (**F**) CFE in 3D organoid cultures of AT2 cells from healthy lungs treated with the NAMPT inhibitor FK866 at different concentrations or with vehicle control (*n* = 3/group; ***P < 0.001, ****P < 0.0001, by 1-way ANOVA). Data are shown as the mean ± SEM. All experiments were repeated at least 3 times.

Next, we evaluated the role of NAMPT in regulating AT2 progenitor cell regeneration. Small-molecule NAMPT activators (NAT, NAT-5r, and SBI-797812) were added to 3D organoid cultures of primary AT2 cells freshly isolated from healthy and IPF lungs, and colony forming efficiency (CFE) was determined. NAT (28) increased the CFE of AT2 cells from both healthy and IPF lungs in a dose-dependent manner (Figure 2, A and B). To determine whether NAT was exerting its effect via NAMPT, we generated immortalized human AT2 cell lines from healthy and IPF lungs (66) and knocked out NAMPT (NAMPT^KO^) with CRISPR/Cas9 in healthy AT2 cells (Supplemental Figure 2A). NAT increased the NAD^+^/NADH ratio in control cells but not in cells with NAMPT KO (Supplemental Figure 2B), confirming NAMPT as the molecular target of NAT. Similarly, the other NAMPT activators, SBI-797812 (33) and NAT-5r (28) (Figure 2, C and D), as well as polyphenolic derivative myricitrin (67) (Supplemental Figure 2C), also promoted AT2 renewal.

To determine if NAD^+^ precursors could replicate the effects of NAT, we compared the effects of NR, NMN, and NAM with NAT in 3D organoid cultures. Our results indicated that NAT had the strongest effect on IPF AT2 cell renewal (Figure 2E). In contrast, blocking NAMPT activity with the highly potent and selective NAMPT inhibitor FK866 suppressed the renewal of human AT2 cells (Figure 2F), which further confirmed the role of NAMPT in regulating AT2 progenitor cell function. Additionally, neither NAT nor FK866 had a significant effect on AT2 cell growth (Supplemental Figure 2, D and E).

### NAMPT expression correlates with SOD2 levels and the oxidative stress response in AT2 cells.

**Figure 3:**
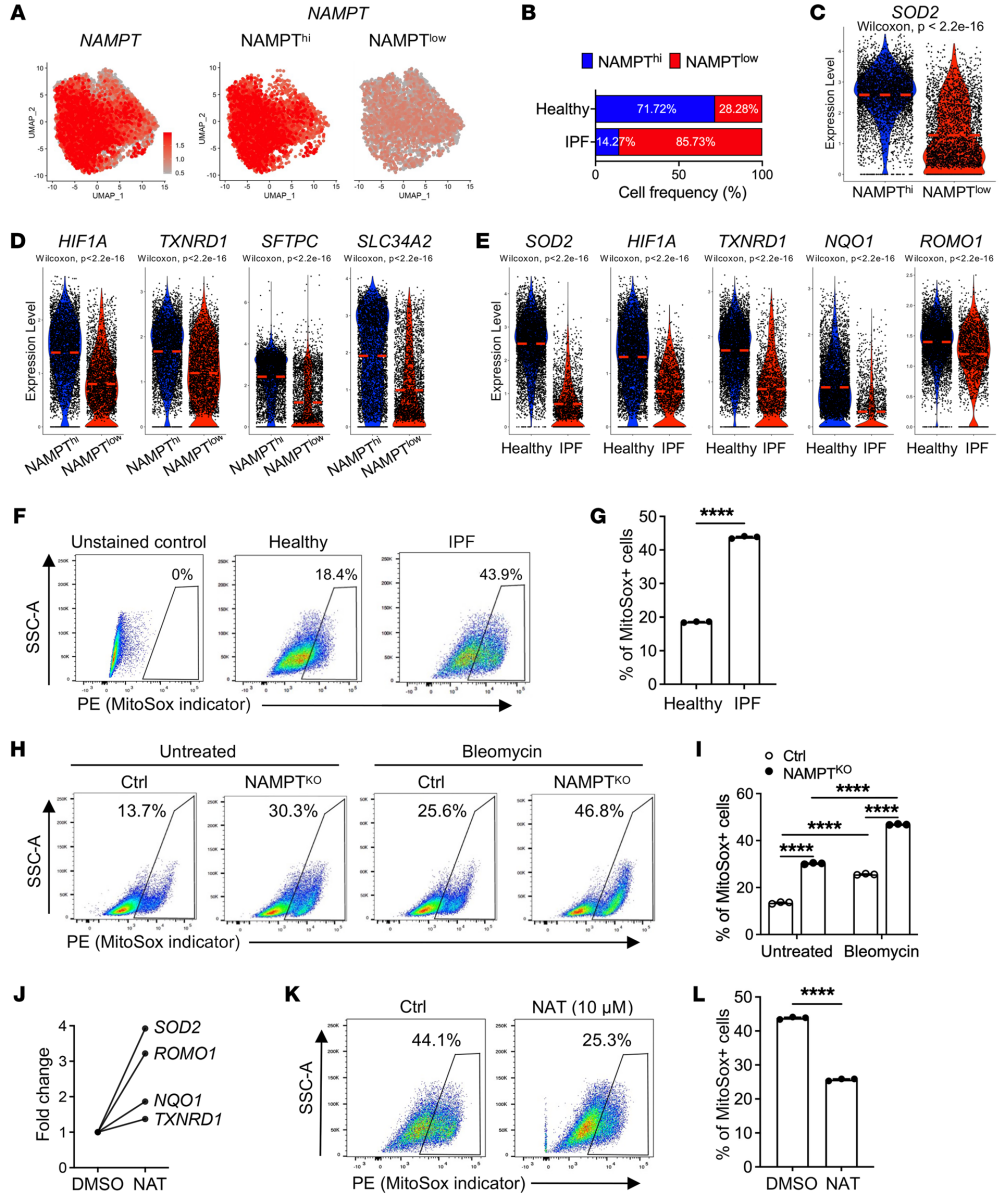
NAMPT is associated with SOD2 expression and the oxidative stress response in AT2 cells. (**A**) UMAP visualization of *NAMPT* expression in total, NAMPT^hi^, and NAMPT^lo^ human AT2 cells. (**B**) Cell frequencies of the NAMPT^hi^ versus NAMPT^lo^ AT2 cells in healthy and IPF lungs. (**C**) Violin plots showing *SOD2* expression levels in NAMPT^hi^ and NAMPT^lo^ human AT2 cells. (**D** and **E**) Violin plots showing expression levels of representative top DEGs of NAMPT^hi^ versus NAMPT^lo^ AT2 cells. (**D**) Expression of *HIF1A*, *TXNRD1*, *SFTPC*, and *SLC34A2* in NAMPT^hi^ and NAMPT^lo^. (**E**) Expression of *SOD2*, *HIF1A*, *TXNRD1*, *NQO1*, and *ROMO1* in healthy and IPF AT2 cells. (**F** and **G**) Flow cytometric analysis of mitochondrial superoxide levels in immortalized AT2 cells from healthy and IPF lungs. (**F**) Gating strategy and (**G**) percentages of mitochondrial superoxide high (MitoSOX^+^) cells (*n* = 3/group; ****P < 0.0001, by unpaired, 2-tailed *S*tudent’s *t* test). (**H** and **I**) Flow cytometric analysis of mitochondrial superoxide levels in NAMPT^KO^ and control immortalized AT2 cells, with and without bleomycin (100 μg/mL) treatment. (**H**) Gating strategy and (**I**) percentages of mitochondrial superoxide high (MitoSOX^+^) cells (*n* = 3/group; ****P < 0.0001, by 2-way ANOVA). (**J**) Fold change in the expression of antioxidant genes in AT2 cells from patients with IPF treated with NAT (10 μM) or DMSO control, analyzed by bulk RNA-seq. (**K** and **L**) Flow cytometric analysis of mitochondrial superoxide levels in immortalized IPF AT2 cells treated with NAT (10 μM) or DMSO control. (**K**) Gating strategy and (**L**) percentages of mitochondrial superoxide^hi^ (MitoSOX^+^) cells (*n* = 3/group; ****P < 0.0001, by unpaired, 2-tailed *S*tudent’s *t* test). Data are shown as the mean ± SEM. All experiments were repeated at least 3 times. SSC-A, side scatter area; PE, phycoerythrin.

To investigate the molecular mechanisms by which NAMPT regulates AT2 progenitor cell function, we separated human AT2 cells from healthy and IPF lungs by their *NAMPT* expression levels: NAMPT high (NAMPT^hi^, expression levels >1) and NAMPT low (NAMPT^lo^, expression levels ≤1) AT2 cells (Figure 3A). In healthy lungs, the majority of AT2 cells (71.72%) were NAMPT^hi^, whereas only a small fraction (14.27%) of AT2 cells from IPF lungs were NAMPT^hi^ (Figure 3B). Differentially expressed gene (DEG) analysis revealed that superoxide dismutase 2 (*SOD2*), a key mitochondrial antioxidant enzyme, was the most downregulated gene in NAMPT^lo^ AT2 cells (Figure 3C). Other oxidative stress–response genes, including *HIF1A* and *TXNRD1*, along with the AT2 marker genes *SLC34A2* and *SFTPC*, were also downregulated in NAMPT^lo^ AT2 cells (Figure 3D).

Importantly, SOD2 and multiple oxidative stress-response genes (68–71), including *NQO1*, *TXNRD1*, and *ROMO1*, were downregulated in AT2 cells from IPF lungs compared with those from healthy controls (Figure 3E). Consistent with these transcriptomics results, we further showed that IPF AT2 cells had increased mitochondrial superoxide levels compared with healthy AT2 cells, as assayed by flow cytometry (Figure 3, F and G).

Next, we looked at the oxidative stress response of human AT2 cells with NAMPT KO (Supplemental Figure 2A). KO of NAMPT increased mitochondrial superoxide levels in AT2 cells both at baseline and after bleomycin treatment (Figure 3, H and I). To further confirm the function of NAMPT in oxidative stress responses in human AT2 cells, we performed bulk RNA-seq analysis of freshly isolated IPF AT2 cells treated with the NAMPT activator NAT for 48 hours. NAT treatment markedly increased the expression of *SOD2* and other oxidative stress-response genes in IPF AT2 cells (Figure 3J). Functionally, NAT treatment also decreased mitochondrial superoxide levels in immortalized IPF AT2 cells (Figure 3, K and L).

These data suggest that NAMPT regulated oxidative stress responses in AT2 cells, with NAMPT deficiency leading to reduced SOD2 expression and increased oxidative stress in IPF AT2 cells, whereas NAMPT activation restored SOD2 level and oxidative balance.

### NAMPT regulates SIRT7 expression and SIRT7 deacetylates SOD2 in AT2 cells.

**Figure 4:**
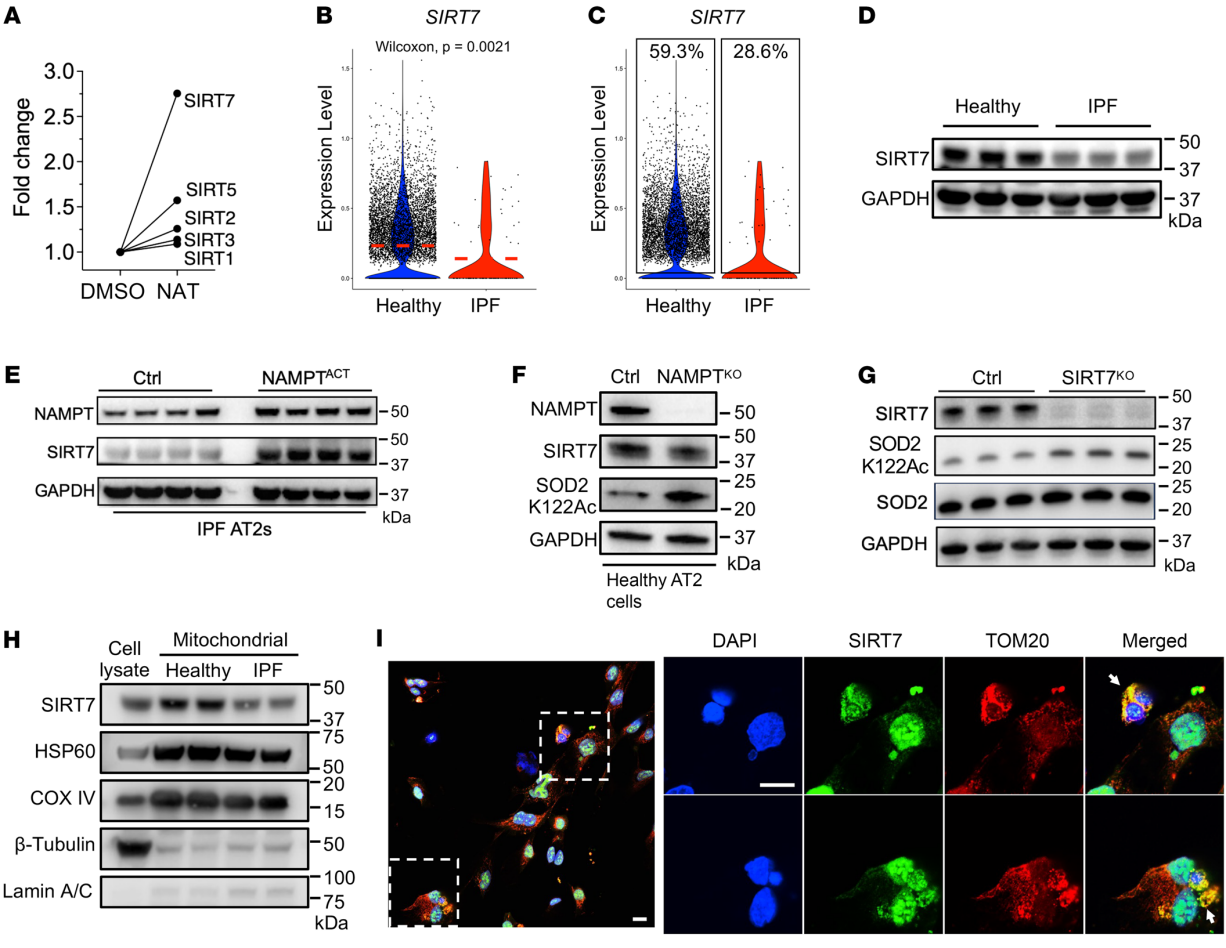
NAMPT regulates SIRT7 expression and SIRT7 deacetylates SOD2 in AT2 cells. (**A**) Fold change in the expression of sirtuin family genes in AT2 cells from patients with IPF treated with NAT (10 μM) or DMSO, analyzed by bulk RNA-seq. (**B** and **C**) Violin plots showing (**B**) *SIRT7* expression levels and (**C**) the percentage of *SIRT7*^+^ cells in AT2 cells from healthy and IPF lungs in the scRNA-seq dataset. (**D**) Western blot analysis of SIRT7 expression in immortalized AT2 cells from healthy and IPF lungs. GAPDH served as a loading control. (**E**) Western blot analysis of the expression of NAMPT and SIRT7 in NAMPT^ACT^ and control immortalized AT2 cells. GAPDH served as a loading control. (**F**) Western blot analysis of the expression of NAMPT, SIRT7, and SOD2-K122Ac in NAMPT^KO^ and control immortalized AT2 cells. GAPDH served as a loading control. (**G**) Western blot analysis of the expression of SIRT7, SOD2-K122Ac, and total SOD2 in SIRT7^KO^ and control immortalized AT2 cells. GAPDH served as loading control. (**H**) Western blot analysis of SIRT7, mitochondrial proteins (HSP60 and COX IV), nuclear protein (lamin A/C), and cytosolic protein (β-tubulin) in mitochondria-isolated fractions from immortalized AT2 cells from healthy and IPF lungs. Whole-cell lysate was used as a control (left lane). (**I**) Immunofluorescence costaining of SIRT7 and TOM20 in immortalized healthy AT2 cells (left). Representative cells (boxed) are shown at higher magnification (right). Arrows indicated the costaining of SIRT7 and TOM20 in boxed cells. Scale bar: 100 μm, low- and high-magnification images). All experiments were repeated at least 3 times.

The enzyme activity of SOD2 is increased by deacetylation of lysine residues 68 (K68) and 122 (K122) situated at the SOD active site (72, 73). SOD2 is deacetylated by the NAD^+^-dependent deacetylases, the sirtuin family proteins (73–75). Using bulk RNA-seq analysis, we found that NAT treatment of freshly isolated IPF AT2 cells dramatically increased *SIRT7* expression (Figure 4A), with modest upregulation of other sirtuins (*SIRT1*, *2*, *3*, and *5*) (Figure 4A). SIRT7 was highly expressed in healthy AT2 cells but significantly downregulated in IPF AT2 cells, both in terms of median expression levels (Figure 4B) and the percentages of *SIRT7*^+^ cells (Figure 4C). We confirmed decreased SIRT7 protein levels in IPF AT2 cells by Western blot analysis (Figure 4D). NAMPT activation (NAMPT^ACT^) in immortalized IPF AT2 cells by CRISPR/Cas9 increased SIRT7 protein levels (Figure 4E), while NAMPT KO in healthy AT2 cells decreased SIRT7 expression and increased K122-acetylation-SOD2 (SOD2-K122Ac, the inactive form of SOD2) (Figure 4F). Furthermore, KO of SIRT7 (SIRT7^KO^) in immortalized human AT2 cells increased SOD2-K122Ac levels without affecting total SOD2 levels (Figure 4G). These data suggested that SIRT7 deacetylated and activated SOD2 in AT2 cells.

SIRT7 is primarily but not exclusively localized to the nucleus (38, 76–78). A recent study showed that SIRT7 protected neural stem cells by suppressing mitochondrial protein folding stress, suggesting that SIRT7 functions in mitochondria (79). To determine whether SIRT7 is localized to mitochondria in AT2 cells, we performed Western blot analysis of mitochondrial fractions from immortalized healthy and IPF AT2 cells. SIRT7 was present along with the classical mitochondrial markers HSP60 and complex IV subunit IV (COX IV) (Figure 4H), providing evidence of its localization within mitochondria. Notably, mitochondrial SIRT7 levels were markedly reduced in IPF AT2 cells compared with healthy AT2 cells (Figure 4H), suggesting that mitochondrial SIRT7 deficiency may contribute to AT2 dysfunction. As expected, immunofluorescence showed predominant nuclear localization of SIRT7, but, importantly, we observed abundant overlap of SIRT7 with the mitochondrial marker TOM20, further supporting its mitochondrial localization (Figure 4I and Supplemental Figure 3).

### NAMPT regulates mitochondrial function and AT2 cell differentiation.

**Figure 5:**
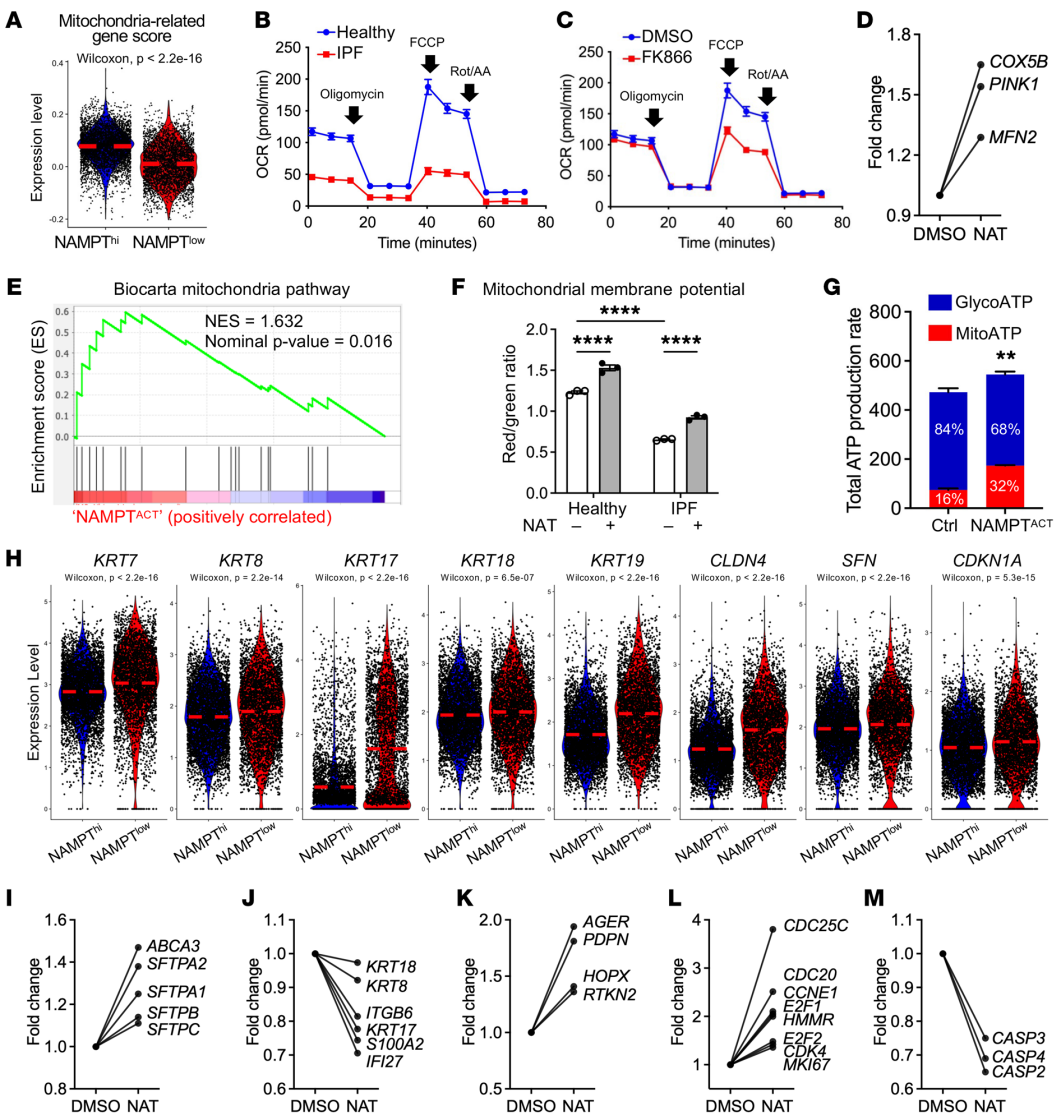
NAMPT regulates mitochondrial function and AT2 cell differentiation. (**A**) Violin plots showing comparison of the mitochondria-related gene scores for NAMPT^hi^ and NAMPT^lo^ human AT2 cells in the scRNA-seq dataset. (**B** and **C**) The OCR of (**B**) immortalized healthy and IPF AT2 cells and (**C**) immortalized healthy AT2 cells treated with FK866 or vehicle control was measured by Seahorse analysis (*n* = 6/group). (**D**) Fold change in the expression of mitochondrial genes in AT2 cells from patients with IPF treated with NAT (10 μM) or DMSO control, analyzed by bulk RNA-seq. (**E**) Enrichment plot for Biocarta mitochondria pathway by gene set enrichment analysis (GSEA) of NAMPT^ACT^ versus control immortalized AT2 cells. Green curves indicate enrichment scores. The normalized enrichment score (NES) and family-wise error rate (FWER) *P* value are indicated in the graph. A NES of greater than 1.1 and a *P* value of less than 0.05 were considered statistically significant. (**F**) Mitochondrial membrane potential of immortalized healthy and IPF AT2 cells treated or not with NAT, evaluated by JC-1 staining followed by flow cytometry (*n* = 3/group; ****P < 0.0001, by 2-way ANOVA). (**G**) Mitochondrial ATP production of immortalized IPF AT2 cells with NAMPT activation by CRISPR/Cas9 and the control was measured by the Seahorse XF Real-Time ATP Rate Assay kit. Data are shown as the mean ± SEM from 6 replicates. **P < 0.01, by unpaired, 2-tailed *S*tudent’s *t* test. (**H**) Violin plots of transitional cell marker genes in NAMPT^hi^ versus NAMPT^lo^ human AT2 cells. (**I**–**M**) Fold change in the expression of (**I**) AT2, (**J**) transitional genes, (**K**) AT1 cell marker genes, (**L**) proliferation genes, and (**M**) caspase genes in AT2 cells from patients with IPF treated with NAT or DMSO, analyzed by bulk RNA-seq. Data are shown as the mean ± SEM. All experiments were repeated at least 3 times. Rot/AA, rotenone/Antimycin A.

Mitochondrial dysfunction impairs postnatal alveolar epithelial cell development (80). To determine whether NAMPT regulates mitochondrial function in AT2 cells, we first compared mitochondria-related gene expression profiles by defining a mitochondrial gene expression score (81) (Supplemental Table 1). As expected, NAMPT^lo^ AT2 cells had a markedly decreased mitochondria-related gene score than did NAMPT^hi^ AT2 cells (Figure 5A). Seahorse mitochondria stress testing revealed decreased oxygen consumption rate (OCR) in IPF AT2 cells compared with healthy controls (Figure 5B). Importantly, inhibition of NAMPT with FK866 decreased the OCR of healthy AT2 cells (Figure 5C), which suggested that NAMPT has a direct role in regulating mitochondrial function. Conversely, NAMPT activation in freshly isolated IPF AT2 cells with NAT treatment increased the expression of mitochondria-related genes including *COX5B*, *PINK1*, and *MFN2* (Figure 5D). Pathway analyses confirmed that NAMPT activation was positively correlated with mitochondrial signaling pathways (Figure 5E). Functionally, NAMPT activation increased mitochondrial membrane potential (Figure 5F), total ATP production (Figure 5G), and ATP production from mitochondrial respiration in AT2 cells (Figure 5G, red bars).

Mitochondrial complex I–driven NAD^+^ regeneration prevents pathologic integrated stress response activation and transitional cell accumulation during alveolar development (80), suggesting that mitochondrial dysfunction may promote transitional cell persistence. Supporting this observation, NAMPT^lo^ AT2 cells also showed increased expression of transitional cell marker genes (82–84), including *KRT8*, *KRT17*, *KRT19*, and *CLDN4* compared with NAMPT^hi^ AT2 cells (Figure 5H). To better visualize this phenotype, we defined a transitional cell score based on the average expression of the transitional cell signature genes (Supplemental Table 1). As previously reported (82), AT2 cells from IPF lungs exhibited significantly elevated transitional cell scores (Supplemental Figure 4A). Consistent with individual marker expression, NAMPT^lo^ AT2 cells also had markedly higher transitional cell scores (Supplemental Figure 4B). These findings suggest that NAMPT deficiency contributes to transitional cell persistence and impaired AT2 differentiation in IPF lungs.

To further define the role of NAMPT in AT2 differentiation, we analyzed bulk RNA-seq on freshly isolated IPF AT2 cells treated with the NAMPT activator NAT. NAT treatment increased the expression of both AT2 and AT1 marker genes (Figure 5, I and K, respectively) while reduced the expression of transitional cell genes (Figure 5J). Meanwhile, genes associated with cell proliferation were significantly upregulated (Figure 5L), whereas apoptosis-related genes were downregulated (Figure 5M). Together, these data suggest that NAMPT activation promotes AT2 cell function and differentiation into AT1 cells and suppresses transitional cell persistence and apoptosis.

### AT2-specific Nampt deletion impairs AT2 progenitor cell renewal in vivo and increases lung fibrosis.

To examine the role of NAMPT in regulating AT2 progenitor cell function and lung fibrosis, we generated a mouse model with AT2-specific *Nampt* deletion (Nampt^AT2^) by cross-breeding *Nampt^fl^* mice (85, 86) with *Sftpc-CreER* mice (Supplemental Figure 5A). NAMPT expression was abrogated in AT2 cells upon tamoxifen treatment (Supplemental Figure 5B). To distinguish the AT2 cells from the Nampt^AT2^ lungs, we performed scRNA-seq on epithelial cells from uninjured lungs and from lungs 4 days after bleomycin injury in Nampt^AT2^ and littermate control mice. On the basis of canonical marker expression (Supplemental Figure 5C), we identified distinct epithelial cell subpopulations, including AT2 and AT1 clusters (Supplemental Figure 5, C and D). Notably, a cluster of transitional cells emerged following bleomycin injury (Supplemental Figure 5, C and D). Importantly, the proportion of these transitional cells was markedly increased in bleomycin-injured Nampt^AT2^ mouse lungs compared with control lungs (Supplemental Figure 5, E and F). Similar to the observations in human NAMPT^lo^ AT2 cells, AT2 cells from Nampt^AT2^ lungs had decreased expression of oxidative stress–response genes, including *Sirt7*, *Sod2*, and *Romo1*, after bleomycin injury (Supplemental Figure 5G). Consistently, Nampt-deficient AT2 cells showed decreased mitochondria-related gene expression scores (Supplemental Figure 5H) and elevated mitochondrial superoxide levels compared with controls (Supplemental Figure 5I). These data further confirmed that NAMPT regulates oxidative stress and mitochondrial function in AT2 cells.

**Figure 6:**
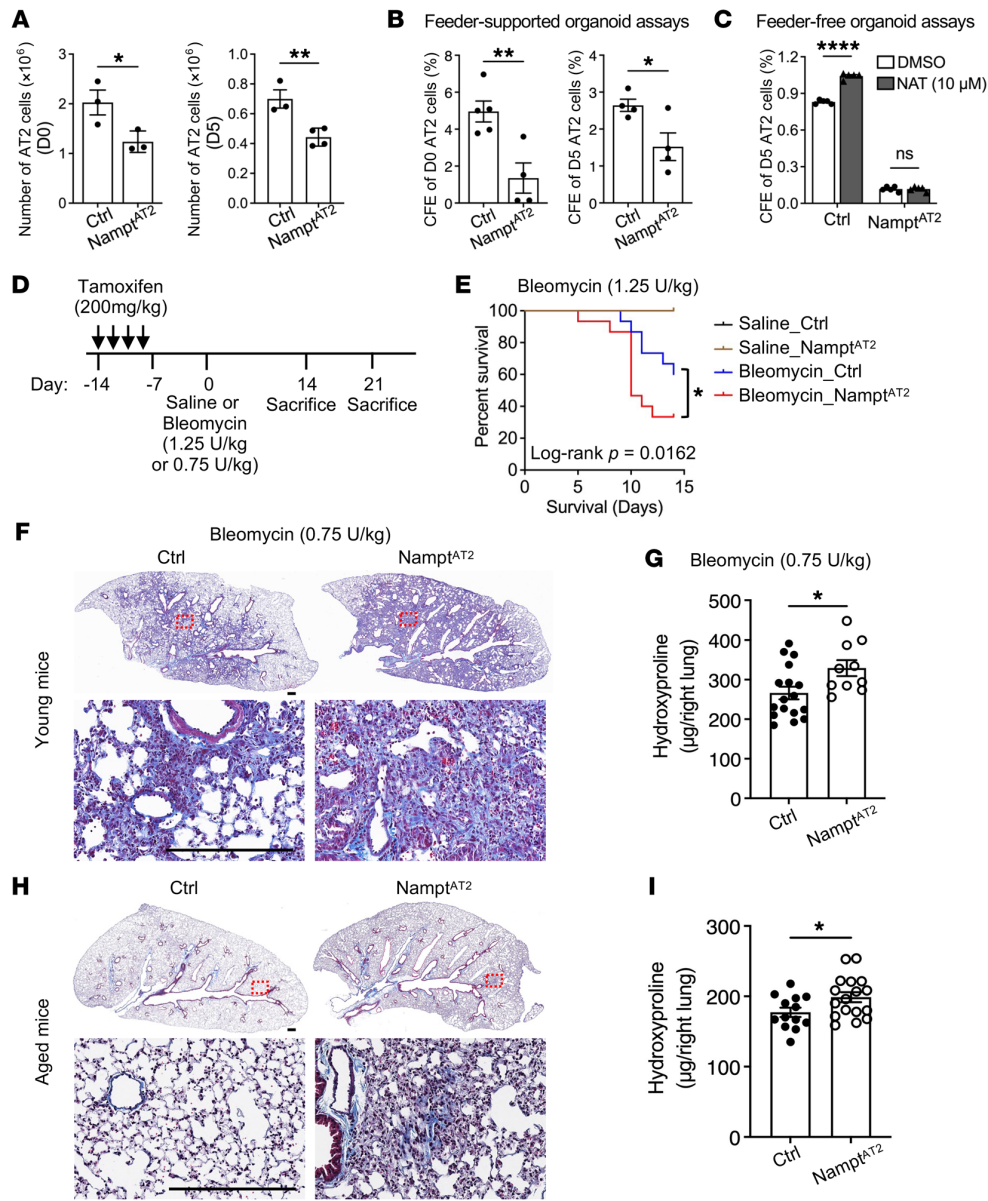
AT2-specific Nampt deletion impairs AT2 progenitor renewal and increases lung fibrosis. (**A**) Number of AT2 cells per lung for uninjured cells (day 0 [D0]) and cells 5 days after bleomycin injury (day 5 [D5]) in Nampt^AT2^ and control mice (*n* = 3–4/group; *P < 0.05, **P < 0.01, by unpaired, 2-tailed *S*tudent’s *t* test). (**B**) CFE in MLg2908 feeder-supported 3D organoid cultures of AT2 cells from Nampt^AT2^ and control mice, uninjured (day 0) or 5 days after bleomycin injury (day 5) (*n* = 4–5/group; *P < 0.05, **P < 0.01, by unpaired, 2-tailed *S*tudent’s *t* test). (**C**) CFE in feeder-free 3D organoid cultures of AT2 cells from Nampt^AT2^ and control mice treated with NAT or DMSO 5 days after bleomycin injury (day 5) (*n* = 5/group; ****P < 0.0001, by 2-way ANOVA). (**D**) Experimental layout for young Nampt^AT2^ and control mice treated with 1.25 U/kg for 14 days for survival curve analysis, or with 0.75 U/kg bleomycin for 21 days for fibrosis evaluation. (**E**) Survival curve (*n* = 15/group; *P < 0.05, by log-rank (Mantel-Cox) test) for young Nampt^AT2^ and control mice treated with 1.25 U/kg bleomycin. (**F**) Trichrome staining and (**G**) hydroxyproline contents in the lungs of young Nampt^AT2^ and control mice treated with 0.75 U/kg bleomycin and sacrificed on day 21 (control, *n* = 17, Nampt^AT2^, *n* = 10; *P < 0.05, by unpaired, 2-tailed *S*tudent’s *t* test). Scale bar: 500 μm. (**H**) Trichrome staining and (**I**) Hydroxyproline contents in lungs of Nampt^AT2^ mice at the age of 14 months (control, *n* = 13, Nampt^AT2^, *n* = 17; *P < 0.05, by unpaired, 2-tailed *S*tudent’s *t* test). Scale bars: 500 μm. Boxed areas are shown at higher magnification (**F** and **H**). Scale bars: 500 μm (low-and high-magnification images). Data are shown as the mean ± SEM. All experiments were repeated at least 3 times.

To further assess the effect of NAMPT deficiency on AT2 cell maintenance after injury, we quantified AT2 cells in the lungs of tamoxifen-treated Nampt^AT2^ and control mice at baseline (day 0) and 5 days after bleomycin injury (day 5) by flow cytometry and cell counting. As expected, bleomycin treatment decreased AT2 cell numbers in both groups (Figure 6A). Notably, tamoxifen-treated Nampt^AT2^ mice had fewer AT2 cells than did controls before (day 0) and 5 days after (day 5) bleomycin treatment (Figure 6A). A decrease in AT2 cells in bleomycin-injured NAMPT^AT2^ lungs was further confirmed by decreased percentages of SFTPC^+^ AT2 cells in immunostained lung sections (Supplemental Figure 5, J and K).

We next assessed AT2 progenitor cell capacity from Nampt^AT2^ mice using 3D organoid assays. AT2 cells isolated from Nampt^AT2^ mice, both from day 0 and from day 5, displayed significantly reduced CFE compared with control cells in feeder-supported culture (Figure 6B and Supplemental Figure 5L). To eliminate any confounding effects of fibroblasts in the feeder-supported assay, we used a feeder-free 3D organoid assay (87) on AT2 cells from day 5 bleomycin-injured Nampt^AT2^ and control mice with NAT or DMSO control. Consistent with the results in the feeder-supported 3D organoid assays, Nampt-deficient AT2 cells showed a pronounced decrease in CFE. NAT significantly enhanced CFE in AT2 cells from control lungs, but not AT2s from Nampt^AT2^ mice (Figure 6C and Supplemental Figure 5M), indicating that NAMPT is essential for maintaining AT2 progenitor cell function following lung injury.

To study the role of NAMPT in bleomycin-induced lung fibrosis in vivo, we treated Nampt^AT2^ and control mice with bleomycin (1.25 U/kg) two weeks after 4 doses of tamoxifen (Figure 6D). Nampt^AT2^ mice showed increased mortality by day 14 compared with controls (Figure 6E). To increase the survival of mice for fibrosis assessment at day 21, we next reduced the bleomycin dose to 0.75 U/kg (Figure 6D). At this dose, Nampt^AT2^ mice showed increased lung fibrosis compared with controls, as evidenced by enhanced pulmonary trichrome staining (Figure 6F) and elevated hydroxyproline content (Figure 6G). Given that NAMPT deficiency contributes to transitional cell accumulation following bleomycin-induced injury (Supplemental Figure 5F), we examined transitional cells using immunostaining for KRT8 and CLDN4. In uninjured lungs, only mild KRT8 expression was observed, primarily in SFTPC^+^ AT2 cells, in both Nampt^AT2^ and control mice. In contrast, fibrotic lungs exhibited markedly increased KRT8 staining, with Nampt^AT2^ lungs showing a dramatic elevation (Supplemental Figure 6, A and B). CLDN4, a more specific marker of transitional cells, was rarely detected in uninjured lungs but was strongly upregulated in fibrotic lungs, with Nampt^AT2^ mice showing significantly higher levels than controls (Supplemental Figure 6, C and D).

We next investigated whether progressive NAMPT deficiency with aging contributes to the development of lung pathology. To test this, young Nampt^AT2^ mice received 4 initial doses of tamoxifen, followed by 2 additional doses of tamoxifen per month until 14 months of age. Intriguingly, the Nampt^AT2^ mice displayed increased collagen accumulation, as shown by both increased trichrome staining (Figure 6H) and significantly elevated hydroxyproline content in the lungs (Figure 6I), suggesting a potential spontaneous fibrosis. Consistently, fibronectin staining (a marker of matrix deposition) was markedly increased in aged Nampt^AT2^ lungs, particularly in regions of heightened cellularity (Supplemental Figure 6E).

These observations revealed that NAMPT deficiency impaired AT2 regeneration, exacerbated bleomycin-induced fibrosis and transitional cell accumulation in young mice, and led to increased collagen deposition in aged mice. Together, these findings underscore the essential role of NAMPT in alveolar repair and long-term lung homeostasis and support its relevance as a major driver of fibrotic lung disease.

### NAMPT activation in vivo promotes AT2 regeneration and attenuated lung fibrosis.

**Figure 7:**
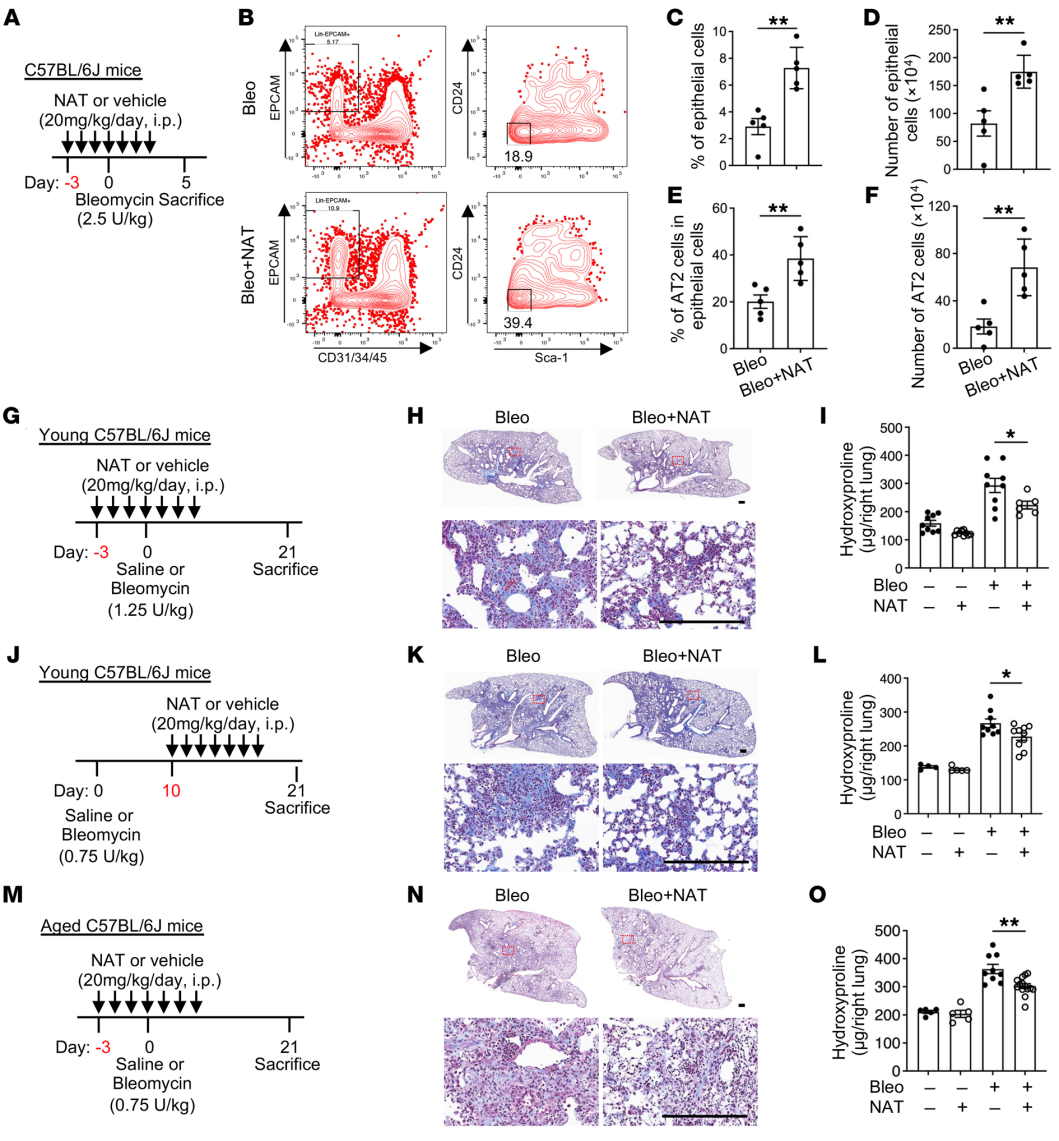
NAMPT activation in vivo promotes AT2 regeneration and attenuates lung fibrosis. (**A**) Experimental layout: 10- to 12-week old (young) C57BL/6J mice were treated with 20 mg/kg NAT and 2.5 U/kg bleomycin, and lungs were collected on day 5. (**B**) Flow cytometric gating strategies for lung epithelial and AT2 cells. Epithelial cells were gated as EPCAM^+^CD31^–^CD34^–^CD45^–^ cells among live cells and AT2 cells as CD24^–^Sca-1^–^ cells within epithelial cell populations. (**C**–**F**) Percentages and total cell numbers of epithelial and AT2 cells per lung (*n* = 5/group; **P < 0.01, by unpaired, 2-tailed *S*tudent’s *t* test). (**G**) Experimental layout: 10- to 12-week-old (young) C57BL/6J mice were treated with 20 mg/kg NAT (from day –3 to day 3) and 1.25 U/kg bleomycin (day 0), and lungs were collected on day 21. (**H**) Trichrome staining (scale bars: 500 μm) and (**I**) hydroxyproline contents (*n* = 6–10/group; *P < 0.05, by ANOVA) were used to assess collagen deposition. (**J**) Experimental layout: 10- to 12-week-old (young) C57BL/6J mice were administrated 0.75 U/kg bleomycin (day 0) and treated with 20 mg/kg NAT at the fibrotic stage (days 10–16), and lungs were collected on day 21. (**K**) Trichrome staining (scale bar: 500 μm) and (**L**) hydroxyproline in the lungs (*n* = 4–10/group; *P < 0.05, by 1-way ANOVA) were used to assess fibrosis. (**M**) Experimental layout: Aged C57BL/6J mice were treated with 20 mg/kg NAT (from day –3 to day 3) and 0.75 U/kg bleomycin (day 0), and lungs were collected on day 21. (**N**) Trichrome staining (scale bar: 500 μm) and (**O**) hydroxyproline in the lungs (*n* = 5–14/group; **P < 0.01, by 1-way ANOVA) were used to assess fibrosis. Boxed areas are shown at higher magnification, scale bars: 500 μm (**H**, **K**, and **N**). Data are shown as the mean ± SEM. All experiments were repeated at least 3 times. Bleo, bleomycin.

Given the critical role of Nampt in maintaining AT2 progenitor cell function, we next investigated whether in vivo NAMPT activation enhances AT2 progenitor cell activity. Young C57BL/6J WT mice were administered NAT to activate NAMPT in vivo. The first treatment paradigm (Figure 7A) involved dosing of NAT (20 mg/kg) via i.p. injection daily from 3 days before to 3 days after bleomycin (2.5 U/kg) treatment. Control mice received bleomycin and vehicle treatment. Mice were sacrificed on day 5 after injury, and lung epithelial cells were isolated, analyzed by flow cytometry, and quantified by cell counting. NAT treatment significantly increased both the percentage (Figure 7, B and C) and total number (Figure 7D) of lung epithelial cells in mice after bleomycin injury. Within the lung epithelial cells, mice treated with NAT showed an increased percentage and number of AT2 cells recovered per lung (Figure 7, B, E, and F). The increase in AT2 cells following NAT treatment in bleomycin-injured lungs was further confirmed by a higher percentage of SFTPC^+^ AT2 cells on lung sections by immunostaining (Supplemental Figure 7, A and B). Interestingly, NAT treatment did not alter the number of total BAL cells or BAL-localized macrophages on day 5 (Supplemental Figure 7, C and D), indicating that NAT treatment did not promote lung inflammation.

The second approach was designed to evaluate a prophylactic effect of NAT in a treatment model, in which the young C57BL/6J WT mice were treated with NAT (20 mg/kg) daily from 3 days before to 3 days after bleomycin administration (1.25 U/kg), and the mice were sacrificed on day 21 (Figure 7G). NAT pretreatment reduced fibrosis as assessed by trichrome staining (Figure 7H) and hydroxyproline levels (Figure 7I) compared with bleomycin alone. We further examined the role of NAT treatment on the transitional cell state of lung epithelial cells by immunostaining. Bleomycin injury induced KRT8 and CLDN4 in fibrotic lungs compared with uninjured lungs, whereas NAT pretreatment significantly lowered the expression of both KRT8 and CLDN4 compared with bleomycin alone (Supplemental Figure 7, E–H), suggesting that NAMPT activation attenuated transitional cell accumulation.

For our third attempt to evaluate the potential therapeutic efficacy of NAT in the bleomycin-induced fibrosis model (Figure 7J), young C57BL/6J WT mice received bleomycin followed by daily administration of 20 mg/kg NAT or vehicle starting on day 10 after bleomycin treatment (when lung fibrosis was well developed) for 7 days (Figure 7J). Mice were sacrificed on day 21, and their lungs were evaluated for fibrosis. Lungs from bleomycin-treated mice dosed with NAT displayed decreased trichrome staining (Figure 7K) and hydroxyproline levels (Figure 7L) compared with mice treated with bleomycin alone, indicating that NAMPT activation by NAT was efficacious for both the prevention and treatment of lung fibrosis in the murine bleomycin model.

IPF is an aging-associated lung disease with a dramatic increase in incidence in patients over 65 years of age. NAD^+^ levels decrease with aging (18, 19, 24). Hence, we tested both the prophylactic and therapeutic effects of NAT on bleomycin-induced lung fibrosis in aged mice. NAT (20 mg/kg/day) or vehicle was administrated either from 3 days before to 3 days after bleomycin injury (Figure 7M), or from day 10 to day 16 after injury (Supplemental Figure 7I). Lungs harvested on day 21 showed that both prophylactic (Figure 7, N and O) and therapeutic (Supplemental Figure 7J) NAT treatment reduced bleomycin-induced lung fibrosis in aged mice, as evidenced by decreased trichrome staining and hydroxyproline levels in the lungs.

These results demonstrate that NAMPT activation by NAT markedly improved lung epithelial cell recovery, increased AT2 cell numbers, and attenuated transitional cell accumulation in mice following bleomycin injury, without promoting inflammation. Both prophylactic and therapeutic NAT administration reduced lung fibrosis in both young and aged mice, demonstrating the therapeutic potential of NAMPT activation in pulmonary fibrosis.

### AT2-specific Sirt7 deletion abolishes AT2 regeneration and fibrosis attenuation.

**Figure 8:**
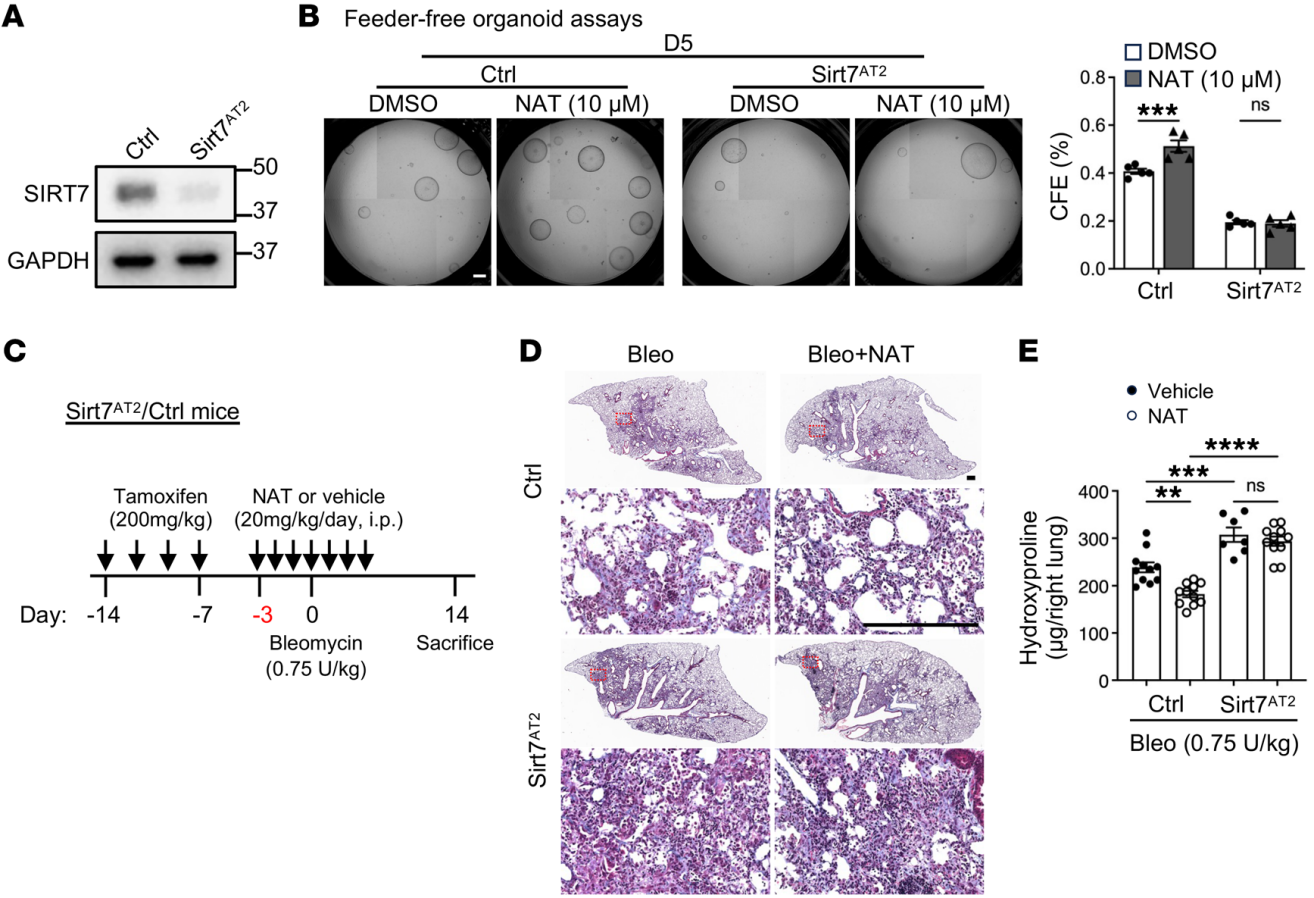
The effects of NAMPT activation on AT2 regeneration and lung fibrosis are abolished in AT2-specific, Sirt7-deleted mice. (**A**) Western blot analysis of SIRT7 expression in control and Sirt7^AT2^ AT2 cells. GAPDH served as a loading control. (**B**) Representative images and CFE of feeder-free 3D organoid cultures of AT2 cells from Sirt7^AT2^ and control mice 5 days after bleomycin injury (*n* = 5/group; ***P < 0.001, by 1-way ANOVA). Scale bar: 300 μm. (**C**) Experimental layout of young Sirt7^AT2^ and control mice treated with 20 mg/kg NAT or vehicle (from day –3 to day 3) and treated with 0.75 U/kg bleomycin (day 0) for 14 days for fibrosis evaluation. (**D**) Trichrome staining (scale bar: 500 μm, low- and high magnification images) and (**E**) hydroxyproline content (*n* = 7–11/group; **P < 0.01, ***P < 0.001, ****P < 0.0001, by 1-way ANOVA) were used to assess collagen levels in mouse lungs. Data are shown as the mean ± SEM. All experiments were repeated at least 3 times.

Here, we demonstrated that NAMPT regulates the SIRT7/SOD2 axis, thereby increasing AT2 cell vulnerability to oxidative stress and mitochondrial dysfunction, ultimately impairing AT2 regenerative capacity and exacerbating lung fibrosis. To further validate this mechanism, we generated mice with AT2-specific deletion of Sirt7 (Sirt7^AT2^), produced by crossing *Sirt7^fl^* mice (38) with *Sftpc-CreER* mice (Figure 8A). We first assessed the progenitor cell capacity of AT2 cells from Sirt7^AT2^ mice using a feeder-free 3D organoid assay. AT2 cells were isolated from Sirt7^AT2^ mice on day 5 following bleomycin injury and cultured in the presence of NAT or vehicle. Sirt7-deficient AT2 cells formed significantly fewer organoids compared with controls, and, importantly, NAT treatment did not rescue CFE in these cells (Figure 8B). These findings indicate that the regenerative effect of NAMPT activation was abolished in the absence of SIRT7.

To determine whether this mechanism influences fibrosis progression, Sirt7^AT2^ and control mice received NAT or vehicle from 3 days before to 3 days after bleomycin administration (0.75 U/kg) (Figure 8C) and were sacrificed on day 14. Consistent with our in vitro findings, bleomycin-challenged Sirt7^AT2^ mice developed more severe fibrosis than did control mice, as shown by increased trichrome staining and hydroxyproline levels. Notably, while NAT treatment reduced fibrosis in control mice, it had no detectable protective effect in Sirt7^AT2^ mice (Figure 8, D and E). Collectively, these results demonstrate that NAMPT-induced protection was dependent on SIRT7 in AT2 cells. Loss of SIRT7 abolished both AT2 regenerative capacity and NAMPT-mediated fibrosis attenuation, supporting a critical role for the NAMPT/SIRT7/SOD2 axis in maintaining AT2 progenitor cell function during lung injury and repair.

## Discussion

Multiple studies from our group and others point to AT2 progenitor cell failure as a critical event in IPF (6, 8, 16, 21, 22), but the molecular mechanisms that regulate AT2 progenitor cell renewal in lung fibrosis are not completely understood. Our discovery of a pronounced reduction of NAMPT in IPF AT2 progenitor cells led us to hypothesize that NAMPT deficiency increases AT2 susceptibility to oxidative stress and mitochondrial dysfunction, resulting in impaired AT2 cell renewal and severe lung fibrosis. NAD^+^ is a central regulator of cell energy metabolism (18, 21), and its levels were found to be decreased in IPF lungs (21, 22). NAD^+^ deficiency is associated with lung fibrosis (21–23). However, the causes of NAD^+^ deficiency in AT2 cells from fibrotic lungs have yet to be elucidated. Here, we have determined that NAMPT activity is crucial for maintaining NAD^+^ levels and AT2 progenitor cell renewal. Inhibition of NAMPT suppressed and activation of NAMPT by small-molecule activators increased renewal of both healthy and IPF AT2 cells in 3D organoid cultures. We further dissected the mechanisms by which NAMPT regulates oxidative stress and mitochondrial function. NAMPT deficiency in IPF AT2 cells downregulates SIRT7 and SOD2, which leads to increased susceptibility of AT2 cells to oxidative stress and mitochondrial dysfunction, resulting in impaired AT2 cell renewal and severe lung fibrosis. Most important, restoration of NAMPT activity by treatment with small-molecule NAMPT activators increased AT2 recovery and attenuated bleomycin-induced lung fibrosis in vivo in both young and aged mice. This work provides mechanistic evidence and highlights potential avenues for the development of new therapies for severe pulmonary diseases such as IPF.

Expression of oxidative stress–response proteins is reduced in IPF epithelial cells (53–55), and the ensuing loss of cellular redox homeostasis has been suggested to be a fundamental driver of IPF pathogenesis for decades (51, 88). Decreased SOD2 in IPF bronchoalveolar lavage (BAL) has led to clinical efforts to restore antioxidant capacity (89). However, broad antioxidant therapies have failed clinically (90, 91), probably due to nonspecific ROS scavenging that disrupts essential redox signaling required for epithelial repair. In contrast, NAMPT activation restores endogenous antioxidant defense by maintaining NAD^+^-dependent SIRT7/SOD2 activity within AT2 cells, preserving mitochondrial homeostasis, oxidative stress resilience, and epithelial regeneration. This cell-intrinsic, mitochondria-focused mechanism may explain why NAMPT-targeted modulation of the SIRT7/SOD2 pathway could succeed where broad antioxidant strategies have not. In the current study, we found that NAMPT deficiency was associated with downregulation of SOD2 in AT2 cells, which resulted in their increased susceptibility to oxidative stress and impaired progenitor cell renewal. This provides a critical missing link identifying the underlying defect in oxidative stress responses in IPF as being related to NAD^+^ metabolism.

We have previously reported that downregulation of NAD^+^-dependent sirtuin signaling contributes to impaired progenitor cell function of IPF AT2 cells and NAD^+^ precursors promote AT2 renewal (8), leading to an ongoing clinical trial involving patients with IPF (NCT06567717). Our current results show that NAMPT activation dramatically upregulated SIRT7 expression, while the expression of other sirtuin family member genes were increased moderately. KO of NAMPT in human AT2 cells decreased SIRT7 expression and SOD2 activation. SIRT7 primarily resides in the nucleus (76, 78). However, the presence of SIRT7 in extranuclear locations has been demonstrated previously (38, 76, 77). In this study, we provide evidence by both Western blotting and immunofluorescence staining that SIRT7 was also present in mitochondria in AT2 cells. SIRT7 deletion increased SOD2-K122Ac, and we further show that SIRT7 deacetylated and activated SOD2 in AT2 cells. As all sirtuins are NAD^+^ dependent, other sirtuin members may also participate in NAMPT activation–associated SOD2 activation, even though we have found that NAMPT activation upregulated SIRT7 most significantly. For example, SIRT3 has been identified as a deacetylase for SOD2 in lung epithelial cells and other cell types (57, 74).

The fundamental role of the lung is to facilitate gas exchange in the distal alveolar space, and this requires the generation of oxygen-transmitting AT1 cells from surfactant-producing AT2 cells. Patients with IPF die from hypoxemia because of loss of AT2 and AT1 cells and failure of oxygen transfer. Impaired AT2-to-AT1 differentiation and accumulation of pathologic transitional epithelial cells have been identified in patients with IPF and mice with experimental lung fibrosis by multiple groups, but the mechanism has been unknown (62, 82–84). Interestingly, we observed increased expression of transitional epithelial markers in NAMPT^lo^ human AT2 cells, suggesting that NAMPT deficiency may impair AT2-to-AT1 differentiation, a focus of ongoing studies. IPF AT2 cells exhibit short telomeres, which correlate with reduced regenerative capacity and mortality (92). Studies are ongoing to determine if there is a link between NAMPT deficiency and telomere shortening.

NAMPT is also expressed in fibroblasts. Published work showed that NAD^+^ metabolism plays a role in regulating lung fibroblast phenotype and apoptosis (93). Our scRNA-seq analysis showed that NAMPT expression levels were not significantly changed in fibroblasts from bleomycin-injured mouse and IPF lungs. More studies on the role of NAMPT in AT2 cell interactions with macrophages and fibroblasts in lung fibrosis are needed in the future.

In this study, we discovered that IPF AT2 cells had reduced expression of cellular NAMPT. NAMPT deficiency downregulated SIRT7 and SOD2 expression, which resulted in increased oxidative stress, mitochondrial dysfunction, and impaired AT2 progenitor cell renewal in IPF. These observations from end-stage IPF explants informed the development of an AT2-specific Nampt-KO mouse model (Nampt^AT2^). These mice had decreased AT2 renewal and reduced recovery from lung injury, decreased SIRT7 and SOD2 expression, increased mitochondrial superoxide, worse survival, increased lung fibrosis after bleomycin injury, and increased collagen accumulation with age, recapitulating many key aspects of severe pulmonary fibrosis in human disease. Excitingly, small-molecule NAMPT activators not only promoted AT2 renewal in 3D organoid cultures, but also attenuated bleomycin-induced lung fibrosis in vivo, with both prophylactic and therapeutic benefits. Specifically, early administration of NAT likely attenuated bleomycin-induced injury by suppressing mitochondrial ROS production, limiting initial epithelial damage and promoting rapid AT2 recovery, whereas delayed NAT administration during the repair phase enhanced AT2 stem cell function, thereby promoting epithelial regeneration and fibrosis resolution. These results indicate that we have discovered the “Achilles heel” of IPF and provide important insights for the development of therapeutics for the fatal disease IPF.

One limitation of our study is the technical difficulty in flow sorting a sufficient number of AT2 cells to measure NAD^+^ levels, given their reduced abundance in IPF lungs. To address this, we generated immortalized human AT2 cells from healthy and IPF lungs to assess NAT effects on NAD^+^ biosynthesis. We acknowledge that these cells may have exhibited some different features and signatures from primary or in vivo AT2 cells due to 2D-culture and immortalization processes (66). These cells were primarily used to investigate molecular mechanisms, including genetic KO and overexpression studies, rather than for detailed analyses of AT2 cell biology or phenotype maintenance. We did not detect a distinct aberrant basaloid cell population in our scRNA-seq dataset and acknowledge that low NAMPT expression levels in such cells could theoretically influence the IPF AT2 phenotype. To address this issue, we confirmed robust expression of canonical AT2 markers (SFTPC, ABCA3) with no detectable KRT5 or KRT17 in the AT2 cell population. Additionally, AT2 cell isolation using HTII-280 may include minor contributions from other epithelial cell populations (<1% basaloid cells) (94). To mitigate this, our immunohistology analyses were restricted to morphologically defined HTII-280^+^ AT2 cells. We used Sca-1 as a negative selection marker for AT2 cells, consistent with our prior work (6), the CD24^–^Sca-1^–^ epithelial cell population represents relatively normal AT2 cells that have recovered from injury, whereas the CD24^–^Sca-1^+^ cell population emerges following bleomycin-induced injury and marks a subset of injured epithelial cells. In this study, treatment with the NAMPT activator NAT increased the percentage of CD24^–^Sca-1^–^ AT2 cells, while reducing the CD24^–^Sca-1^+^ cell population, indicating enhanced recovery and maintenance of healthy AT2 cells. Although the role of NAMPT in these subsets remains to be fully elucidated, this will be addressed in future experiments. We did not examine the role of extracellular NAMPT (eNAMPT), which has been link to inflammation in acute lung injury through effects on endothelial cells and macrophages (86, 95–105). Our results indicated that NAT treatment enhanced AT2 recovery, with no significant changes in lung inflammation. The effect of NAT on eNAMPT expression will be investigated in future studies.

## Methods

### Sex as biological variable.

Both males and females were included in all human and mouse studies, and similar findings are reported for both sexes in all experiments.

### Mice.

*SFTPC-CreER* mice were described previously (13). Nampt-floxed mice (strain no. 034242, RRID: IMSR_JAX:034242) (85) and Sirt7-floxed mice (strain no. 033453, RRID: IMSR_JAX no. 033453) (38) were purchase from The Jackson Laboratory. Nampt^AT2^ and Sirt7^AT2^ mice were generated by crossbreeding *SFTPC-CreER* mice with *Nampt*-floxed or *Sirt7*-floxed mice, respectively. Young (8–10 weeks old) and aged (20–24 months old) C57BL/6J mice were obtained from The Jackson Laboratory and the National Institute on Aging, respectively, and ere housed for at least 2 weeks before experiments. Animals were randomly assigned to treatment groups and were age and sex matched.

### Bleomycin instillation and bronchoalveolar lavage.

Intratracheal bleomycin instillation was performed by following the standard procedure described previously (8, 106). Detailed methods are available in Supplemental Methods.

### Human and mouse lung tissue dissociation.

Human lung single-cell isolation and flow cytometry were performed as previously described (6). In brief, human lung tissues were minced and then digested with 2 mg/mL dispase II, followed by 10 U/mL elastase and 100 U/mL DNase I digestion. Finally, cells were filtered through a 100 μm cell strainer and lysed with RBC lysis to get a single-cell suspension. Detailed methods are provided in Supplemental Methods.

### Immortalized human AT2 cells.

We generated immortalized AT2 cell lines from healthy and IPF human lungs, following the approach established by Offringa’s group (66). Detailed methods are provided in Supplemental Methods.

### 3D organoid cultures of human and mouse AT2 cells.

Flow-sorted AT2 cells were cultured in Matrigel-medium mixture with 2 different protocols in the presence (6, 8) or absence (87) of MLg2908 lung fibroblasts as feeders. Detailed methods can be found in Supplemental Methods.

### Seahorse metabolic flux measurements.

The Seahorse XF Cell Mito Stress Test (Agilent Technologies, 103015-100) and the XF Real-Time ATP Rate Assay Kit (Agilent Technologies, 103592-100) were used to assess the OCR and measure total ATP production rates in living cells, respectively, following the manufacturer’s instructions. Briefly, 1 × 10^4^ cells were seeded on Seahorse XF microplates. One hour before analysis, the culture medium was replaced with Seahorse XF assay media (Agilent Technologies, 103575-100), and plates were incubated at 37°C in a CO_2_-free incubator. Detailed methods are given in Supplemental Methods.

### Mitochondrial ROS measurement.

Mitochondrial ROS production was assessed using the red MitoSOX Superoxide indicator (Thermo Fisher Scientific, M36008), which detects ROS content specifically targeted to mitochondria. Detailed methods are provided in Supplemental Methods.

### NAD/NADH measurement.

Total NAD and NADH were measured by NAD/NADH Quantification Kit (MilliporeSigma, Catalog MAK037) following the manufacturer’s instructions. Detailed methods are in Supplemental Methods.

### Mitochondrial membrane potential measurement.

Mitochondrial membrane potential (MMP) was assessed in control and NAT-treated AT2 cells using the JC-1 probe (Thermo Fisher Scientific, catalog M34152). Cells were stained with 2 μM JC-1 following a previously established protocol (107) and analyzed by flow cytometry. To validate the JC-1 results, 50 μM CCCP staining was also performed.

### scRNA-seq data analysis.

The human lung epithelial scRNA-seq dataset GSE157996, generated by our group (8), along with the datasets GSE146981 (64), GSE128033 (61), GSE135893 (63), GSE136831 (62), GSE122960 (108), and GSE132771 (109) from other groups, were analyzed to assess NAMPT expression in healthy and IPF samples.

The mouse epithelial and immune cell scRNA-seq dataset is under GSE295739. scRNA-seq was performed at the Cedars-Sinai Medical Center Genomics core facility. Lung tissue dissociation, single-cell isolation (6), and 10x Genomics library preparation were performed as described previously (110). Cell Ranger version 7.0.1 (10x Genomics) was used to process raw sequencing data, and Seurat suite version 4.1.0 was used for downstream analysis. Detailed methods including quality control, cell clustering, doublet calling, and annotation were done as previously described (106). DEGs with a log fold change of more than 0.1 between NAMPT^+^ (expression >1) and NAMPT^–^ (expression ≤1) AT2 cells were analyzed in R. Mitochondria-related and transitional gene scores were calculated using the AddModuleScore function based on the average expression levels of the corresponding gene sets (Supplemental Table 1).

### Bulk RNA-seq and data analysis.

Bulk RNA-seq and data analysis were performed as described previously (106). Detailed methods are described in Supplemental Methods.

### Statistics.

Bioinformatics comparisons were calculated using the Wilcoxon signed-rank test. For the scRNA-seq data, the lowest *P* value calculated in Seurat was *P* < 2.2 × 10^–16^. Other data were calculated using GraphPad Prism (GraphPad Software), expressed as the mean ± SEM. No animals were excluded for analysis. All experiments were repeated 3 or more times. Data were normally distributed with similar variance. Two-group comparisons used Student’s 2-tailed *t* test, whereas multiple groups were analyzed by 1-way ANOVA with Bonferroni’s or Dunnett’s post hoc test, or 2-way ANOVA with Tukey’s post hoc test. Significance was set at a *P* value of less than 0.05.

### Study approval.

Human tissue use was approved by the IRBs of Cedars-Sinai Medical Center (Pro00051481) with informed consent obtained from 15 healthy donors and 18 patients with IPF of both sexes. Animal experiments were approved by the IACUC at Cedars-Sinai Medical Center (protocol IACUC008529). All mice were housed in a pathogen-free facility at Cedars-Sinai Medical Center with ad libitum access to water and standard chow.

### Data availability.

The raw data files of the single-cell RNA-seq are deposited in the Gene Expression Omnibus (GEO) database (GEO GSE295739 and GSE295740). The R code files used for data integration and analysis are available at https://github.com/jiang-fibrosis-lab Values for all data points in graphs are reported in the Supporting Data Values file.

## Author contributions

JL, PWN, and DJ conceived the study. XZ performed most of the experiments and analyzed the data. XL analyzed scRNA-seq data and performed experiments. YQ, AR, and NL took part in mouse, cell culturing, and biological experiments. CY analyzed scRNA-seq data. TP and PC provided human samples and interpreted data. CH, BS, DC, and SJG interpreted data and provided comments on the manuscript. XZ, JL, DJ, and PWN wrote the manuscript. All authors reviewed the manuscript.

## Conflict of interest

The authors have declared that no conflict of interest exists.

## Funding support

This work is the result of NIH funding, in whole or in part, and is subject to the NIH Public Access Policy. Through acceptance of this federal funding, the NIH has been given a right to make the work publicly available in PubMed Central.

- NIH grant R01 AG078655 (to JL and PWN).
- NIH grant R35-HL150829 (to PWN).
- NIH grant P01-HL108793 (to PWN).
- NIH grant R01 HL172990 (to DJ).
- American Heart Association Career Development Award 24CDA1268568 (to XL).
- Pulmonary Fibrosis Foundation (PFF) Scholars Program grant 1272558 (to XL).

## Supplementary Material

Supplemental data

Unedited blot and gel images

Supporting data values

## Version 1 05/07/2026

In-Press Preview

## Version 2 06/15/2026

Electronic publication
