# Supplementary material for: Activation of the impaired NAMPT/SIRT7/SOD2 axis restores alveolar progenitor cell renewal in idiopathic pulmonary fibrosis
Source: J Clin Invest. 2026 May 7;136(12):e198031. doi: 10.1172/JCI198031 (PMC13262719; doi:10.1172/JCI198031)
Supplement: Supplemental data [file jci-136-198031-s209.pdf]

Activation of the impaired NAMPT/SIRT7/SOD2 axis restores alveolar progenitor renewal in idiopathic pulmonary fibrosis

Xuexi Zhang,[1] Xue Liu,[1] Yujie Qiao,[1] Anas Rabata,[1] Ningshan Liu,[1] Changfu Yao,[1] Tanyalak Parimon,[1] Danica Chen,[2] Cory Hogaboam,[1] Peter Chen,[1,3] Barry Stripp,[1] Stephen J. Gardell,[4] Dianhua Jiang,[1,3] Paul W. Noble,[5,*] and Jiurong Liang[1,*]

Extended Methods and Supplemental Figures 1 - 7

Supplemental Figure 1: NAMPT expression in epithelial cells and fibroblasts.

Supplemental Figure 2: NAT promotes NAD+ biosynthesis in AT2 cells specifically through NAMPT.

Supplemental Figure 3: Immunofluorescence co-staining of SIRT7 and TOM20 in human AT2 cells.

Supplemental Figure 4: Transitional signatures in AT2 cells.

Supplemental Figure 5: AT2-specific Nampt deletion leads to elevated oxidative stress and transitional cell accumulation after injury.

Supplemental Figure 6: AT2-specific Nampt deletion leads to increased transitional cell accumulation in bleomycin-induced fibrosis model.

Supplemental Figure 7: NAMPT activation *in vivo* promotes AT2 regeneration and reduces transitional cell accumulation in fibrosis model.

Supplemental Table 1. Gene lists used for the gene set scores.

Extended Methods

Human and mouse lung tissue dissociation and flow cytometry

Human lung single cell isolation and flow cytometer were performed as previously described(1). Flow cytometry was performed with Fortessa and BD Symphony S6 Cell Sorter and analyzed with Flow Jo 10.8.1 software (BD Biosciences). Human AT2 cells were sorted as Epcam^{+}CD31^{-} CD45^{-}HTII-280^{+} cells. Anti-human CD31 (clone WM59, Catalog # 303118, RRID AB_2247932), anti-human CD45 (clone WI30, Catalog # 304016, RRID AB_314404), anti-human EpCAM (clone 9C4, Catalog # 324212, RRID AB_756086) were from BioLegend. Anti-HTII-280 (Catalog # TB-27AHT2-280, RRID: AB_2832931) were from Terrace biotech.

Mouse lung tissue dissociation and single cell isolation were described previously (1). In brief, human lung tissues were minced and then digested with 2 mg/ml Dispase II, followed by 10 U/ml elastase and 100 U/ml DNase I digestion. Finally, cells were filtered through 100 μm cell strainer and lysed with red blood cell lysis to get single cell suspension. Mouse AT2 cells were gated as Epcam^{+}CD31^{-}CD34^{-}CD45^{-}CD24^{-}Sca-1^{-} population for flow sorting. FITC anti-mouse CD24 (Clone M1/69, Catalog #101806, RRID: AB_312839), PE/Cyanine7 anti-mouse Ly-6A/E (Sca-1) (Clone D7, Catalog #108114, RRID: AB_493596) and APC/Cyanine7 Streptavidin (Catalog #108114) were from BioLegend. Biotin anti mouse CD31 (PECAM-1) (Clone 390, Catalog # 13-0311-85, RRID: AB_466421), Biotin anti mouse CD45 (Clone 30-F11, Catalog # 13-0451-85, RRID: AB_466447), Biotin anti mouse CD34 (Clone RAM34, Catalog # 13-0341-85, RRID: AB_466426) were from eBioscience.

Cell lines

Mouse lung fibroblast cell line MLg2908 (Catalog CCL-206) was from ATCC.

In vitro culture of immortalized human AT2 cells.

Due to the lack of commercially available cell lines that retain the characteristics of primary human AT2 cells, and the limitations of 2D-culture and genetic manipulation in freshly isolated AT2 cells, we optimized a protocol to generate immortalized AT2 cell lines from healthy and IPF human lungs, following the approach established by Dr. Offringa's

group (2). Briefly, isolated healthy and IPF AT2 cells were resuspended in Fmed + ROCKinh medium, consisting of a 3:1 ratio of DMEM/F12 (Corning, Catalog 10-090-CV) to DMEM (Gibco, Catalog 21063-029), supplemented with 5% FBS, 0.4 mg/mL hydrocortisone (Sigma-Aldrich, Catalog H0888), 5 mg/mL insulin (Sigma-Aldrich, Catalog I0516), 8.4 ng/mL cholera toxin (Sigma-Aldrich, Catalog C8052), 10 ng/mL human recombinant EGF (ThermoFisher, Catalog PHG0311), Antibiotic-Antimycotic (Gibco, Catalog 15240-062), and 10 mM Y-27632 (Enzo Life Sciences, Catalog 270–333). The cells were allowed to attach for two days, with media changes every 2–3 days, and monitored daily for survival and proliferation. Once AT2 cells reached passage 3–4, they were infected with SV40 LgT lentivirus (customized from VectorBuilder) for immortalization, followed by G418 (Sigma-Aldrich, Catalog G8168) antibiotic selection. Single clones were sorted using HTII-280 in 96-well plates, and all AT2 cells were maintained in Fmed + ROCKinh medium.

Generation of NAMPT-KO, NAMPT-activation, SIRT7-KO cell line with CRISPR system

For knock out, immortalized AT2 cells were infected with commercial NAMPT sgRNA CRISPE/Cas9 All-in-One Lentivirus (Abm, Catalog #31414111) or commercial SIRT7 sgRNA CRISPE/Cas9 All-in-One Lentivirus (Abm, Catalog # 43812111). For activation, Immortalized AT2 cells were infected with commercial NAMPT CRISPRa sgRNA lentivirus (Abm, Catalog # 31414121). The efficiency of knock out or activation were validated by western blot.

Bleomycin instillation and bronchoalveolar lavage

Bleomycin instillation was described previously(1). Under anesthesia, the trachea was surgically exposed. 0.75 – 2.5 U/kg bleomycin (Hospira) in 25 µl PBS was instilled into the mouse trachea with a 25-G needle inserted between the cartilaginous rings of the trachea. Control animals received saline alone. The tracheostomy site was sutured, and the animals were allowed to recover. Mice were sacrificed at different time points and lung tissue were collected for experiments. Bronchoalveolar lavage was described previously(3), mice were anesthetized, and lungs and heart were surgically exposed. The trachea was cannulated, and the lungs were lavaged three times with 0.8-ml aliquots of

cold PBS. The first 0.8-ml bronchoalveolar lavage (BAL) was used for cytokine protein measurement. The live cells from all three 0.8-ml aliquots of BAL were recovered and counted using a hemocytometer. Cytospin preparations of BAL cells were stained conventionally, and differential cell counts were performed.

For mice NAT treatment, the compound NAT (MedChemExpress, Catalog HY-144776) was dissolved in DMSO, mixed with four volumes of Kolliphor (Sigma-Aldrich, Catalog C5135), and further diluted with eight volumes of 5% dextrose (Sigma-Aldrich, Catalog D9434) to prepare the working solution(4). Mice received daily intraperitoneal injections of NAT or vehicle at a dose of 20 mg/kg for one week, either starting three days before and continuing three days after bleomycin administration or beginning on Day 10 and continuing for seven consecutive doses following bleomycin treatment.

Hydroxyproline

Collagen contents in mouse lungs were measured with a conventional hydroxyproline method(5). In brief, lung tissues were vacuum dried and hydrolyzed with 6N hydrochloride acid at 120°C for overnight. Hydroxyproline content was measured and expressed as mg per lung. The ability of the assay to completely hydrolyze and recover hydroxyproline from collagen was confirmed using samples containing known amounts of purified collagen.

RNA analysis

RNA was extracted using TRIzol reagent. For real-time PCR analysis, 1 μg total RNA was used for reverse transcription with PrimeScript™ RT Master Mix (Perfect Real Time) (TAKARA). 1.25 μl cDNA was subjected to real-time PCR by using Power SYBR Green PCR Master Mix (Applied Biosystems) and the ABI 7500 fast Real-Time PCR system (Applied Biosystems). The specific primers are listing below: Human *NAMPT* forward GCAGAAGCCGAGTTCAACATC, and reverse TTTTCACGGCATTCAAAGTAGGA.

Western blotting

Proteins were assessed with western blotting as previously described (6). The membrane were probed with antibodies against human NAMPT (Cell Signaling Technology, Catalog

#61122, RRID: AB_2799602, 1:1000), mouse NAMPT (Proteintech, Clone 3D4D8, Catalog #66385-1g, RRID: AB_2881761, 1:1000), human SIRT7 (Santa Cruz, Catolog #sc-365344, RRID: AB_10850175, 1:1000), human SOD2/MnSOD (acetyl K122) (Abcam, Clone NCI-R156-33, Catalog, ab240391, RRID:AB_2892634, 1:1000), human SOD2 (Cell Signaling Technology, Catalog #13194s, RRID: AB_2750869, 1:1000), human HSP60 (ThermoFisher, Catalog #MA3-012, RRID: AB_2121466, 1:1000), human COX IV (Abcam, Catalog #ab202554, RRID: AB_2861351, 1:1000), human Lamin A/C (Cell Signaling Technology, Catalog #2032, RRID: AB_2136278, 1:1000). GAPDH (Cell Signaling Technology, Clone 14C10, Catalog #2118s, RRID: AB_561053, 1:5000) and β-tubulin (GenScript, Clone 2G7D4, Catalog #A01717, RRID: AB_2622210, 1:5000) were used as a loading control.

Histology and immunofluorescence staining

Freshly dissected human and mouse lung tissues were fixed in 10% neutral formalin (Sigma-Aldrich) overnight, dehydrated through an ethanol gradient, and embedded using a tissue embedding machine. Paraffin sections (5 μm thick) were cut using a Microm HM325 microtome, mounted on Superfrost Plus microscope slides, and rehydrated through xylene and an ethanol gradient. Antigen retrieval was performed using citrate-based antigen unmasking solutions before antibody staining. The sections were incubated with anti-NAMPT (Novus, Catalog # NBP2-19467, 1:100), anti-HTII-280 (Terrace Biotech, Catalog # TB-27AHT2-280, RRID: AB_2832931, 1:100), anti-Sftpc (proteintech, Catalog # 10774-1-AP, RRID: AB_2185497, 1:200), anti-Pdpn (DSHB, Catalog # 8.1.1, RRID: AB_3192699,1:50), anti-Krt8 (DSHB, Catalog # TROMA-I, RRID: AB_531826, 1:200), anti-Cldn4 (Thermo Fisher Scientific, Cat # 36-4800, RRID: AB_2533262, 1:200), and anti-Fibronectin (Bio-Rad, Cat # 4470-2750, RRID:AB_620146, 1:200) for overnight at 4°C followed by incubation with secondary antibodies, AF488 goat anti-mouse IgM (Jackson Immunoresearch, Catalog #115-545-020, RRID: AB_2338843), Cy3 goat anti-rabbit IgG (Jackson Immunoresearch, Catalog #111-165-003, RRID: AB_2338000), AF488 goat anti-syrian hamster (Jackson ImmunoResearch, Cat # 107-545-142, RRID: AB_2337478), AF488 goat anti-rat (Thermo Fisher Scientific, Ca t# A-11006, RRID: AB_2534074), and AF488 goat anti-mouse (Jackson ImmunoResearch,

Cat # 115-545-146, RRID: AB_2307324), at 1:400 dilution in blocking buffer for 2h at RT. At least 10 random fields of view per section were photographed (20× objective) on a Zeiss LSM 780 Confocal Microscope (ZEISS). NAMPT signal quantification in HTII-280–positive cells were performed using ZEISS software. Three samples per group were analyzed, with 50–60 individual AT2 cells measured per lung.

For immunofluorescence staining of cells in culture, cells were fixed in 4% paraformaldehyde for 10 min, followed by permeabilization with 0.1% (v/v) Triton-100/PBS for 20 min at RT and blocked with blocking buffer (10%(v/v) goat serum in PBS) at RT for 1h. They were then incubated with primary antibodies, anti-SIRT7 (Santa Cruz, Catalog # sc-365344, RRID: AB_10850175) and anti-TOM20 (Proteintech, Catalog # 11802-1-AP, RRID: AB_2207530), at 1:100 dilution for overnight at 4°C. Subsequently, samples were incubated with secondary antibodies (AF 488 goat anti-mouse IgG (Jackson Immunoresearch, Catalog #115-545-146, RRID: AB_2307324), Cy3 goat anti-rabbit IgG (Jackson Immunoresearch, Catalog #111-165-003, RRID: AB_2338000)) at 1:400 dilution in blocking buffer for 2h at RT. Nuclei were counter-stained with 1 μg/mL DAPI (BD Biosciences, Catalog # 564907, RRID: AB_2869624) for 10 min at RT. Immunofluorescence intensity quantification was performed by Halo image analysis software (v4.0.5, Indica Labs) with the HighPlex FL module (v4.2.3).

3D-organoid cultures of AT2 cells with Mlg2908 as feeders

Flow-sorted human ($EpCAM^{+}HTII\text{-}280^{+}$) or mouse ($EpCAM^{+}CD24^{-}Sca\text{-}1^{-}$) AT2 cells were cultured in a 1:1 Matrigel/medium mixture in the presence of MLg2908 lung fibroblasts. A total of 3×10^{3} AT2 cells and 2×10^{5} MLg2908 cells (Catalog #CCL-206, ATCC) were plated in 100 μl of the Matrigel/medium mixture onto 24-well, 0.4 μm Transwell inserts, with 410 μl of medium added to the lower chamber. Cells were cultured either in medium alone or with specified treatments. The culture medium consisted of DMEM/F12 supplemented with 10% FBS, ITS (insulin-transferrin-selenium), the TGF-β receptor inhibitor SB431542 (10 μM), and antibiotics, as previously described (1). Growth factor–reduced Matrigel (Catalog #354230) was obtained from Corning Life Sciences. The following treatments were applied continuously from the beginning to the end of the organoid culture for NAMPT activation or inhibition: 5 or 10 μM NAT (MedChemExpress,

Catalog HY-144776), 5 or 10 μM NAT-5r (Wuxi AppTec), and 5 or 10 μM SBI-797812 (MedChemExpress, Catalog HY-126255); 0.5, and 2 nM FK866 (Sigma-Aldrich, Catalog 481908); 100 μM nicotinamide mononucleotide (NMN, Sigma-Aldrich, Catalog N3501); and 100 μM nicotinamide riboside (NR, Cayman Chemical, Catalog 23132,). 10 mM nicotinamide (NAM, Sigma-Aldrich, Catalog N0636). The same volume of DMSO served as a control. Fresh medium was changed every other day, and cultures were maintained in a humidified incubator at 37°C with 5% CO_2. Colonies were visualized using an EVOS ™ XL Core Cell Imaging System (Invitrogen). Colony-forming efficiency (CFE) was calculated 12 days after plating by counting colonies ⩾50 μm in diameter per insert and expressing the number as a percentage of input epithelial cells.

Feeder-free 3D-organoid cultures AT2 cells

Feeder-free organoid culture was performed as previously described (7). A total 8×10^3 freshly flow-sorted AT2 cells were plated in 100 μl of a 1:1 Matrigel/medium mixture onto 24-well, 0.4 μm Transwell inserts, with 600 μl of medium added to the lower chamber. Growth factor–reduced Matrigel (Catalog #354230) was obtained from Corning Life Sciences. The Matrigel was allowed to solidify for 30 min at 37 °C. Organoids were cultured in AT2 maintenance medium (7) consisting of 3 μM CHIR99021 (Tocris Bioscience, Catalog 4423), 1 μM BIRB796 (Tocris Bioscience, Catalog 5989), 10 μM SB431542 (Abcam, Catalog Ab120163) 1× Insulin-Transferrin-Selenium (ITS; Thermo, Catalog 41400-45), 15 mM HEPES (Thermo, Catalog 15630080), 1× Anti-Anti (Thermo, Catalog A56955), 1× GlutaMAX (Thermo, Catalog 35050061), 50 ng/mL hEGF (Gibco, Catalog PHG0313), 5 μg/mL Heparin (Sigma-Aldrich, Catalog H3149), 10 ng/mL mNoggin (PeproTech, Catalog 250-38), 1× B27 supplement (Thermo, Catalog 17504044), 1× N2 supplement (Thermo, Catalog 17502048), 1.25 mM N-acetylcysteine (Sigma-Aldrich, Catalog A9165), 10 ng/mL mFGF10 (R&D system, Catalog 751004) and 10 ng/mL mIL-1β (BioLegend, Catalog 575104) in Advanced DMEM/F12 (Thermo, Catalog 12634028). For the first two days, the medium was supplemented with 10 μM ROCK inhibitor (Y-27632 2HCl, Selleckchem, Catalog S1049). Thereafter, the AT2 maintenance medium was replaced every other day for the duration of the experiment. NAMPT activation was induced by continuous treatment with 10 μM NAT (MedChemExpress,

Catalog HY-144776) from the beginning of the culture. Colonies were visualized using an EVOS™ XL Core Cell Imaging System (Invitrogen). Colony-forming efficiency (CFE) was calculated 12 days after plating by counting colonies ⩾50 μm in diameter per insert and expressing the number as a percentage of input epithelial cells.

Bulk RNA-seq and data analysis

Single-cell suspensions were prepared from IPF lung tissues and cultured in serum-free medium supplemented with either NAT (10 μM) or DMSO as a control for 48 hours. Subsequently, AT2 cells were isolated by flow cytometry based on the gating strategy for live $CD31^{-}CD45^{-}EpCAM^{+}HTII\text{-}280^{+}$ cells, and RNA was extracted as previously described. For NAMPT knockout and activation in immortalized AT2 cell lines, RNA was isolated from cells cultured in 60 mm plates. The extracted total RNA was submitted to the Cedars-Sinai Medical Center Genomics Core and the UCLA Technology Center for Genomics & Bioinformatics (TCGB) for library preparation and RNA sequencing. Sequencing was performed on a NovaSeq X Plus platform, generating 30 million reads per sample. Gene Set Enrichment Analysis (GSEA) of differentially expressed genes was conducted using the GSEA too. The raw RNA-seq data generated in this study have been deposited in the NCBI Gene Expression Omnibus (GEO) database. (GEO GSE295740).

Seahorse metabolic flux measurements

The Seahorse XF Cell Mito Stress Test (Agilent Technologies, 103015-100) and Seahorse XF Real-Time ATP Rate Assay Kit (Agilent Technologies, 103592-100) were used to assess oxygen consumption rate (OCR) and measure total ATP production rates in living cells, respectively, following the manufacturer's instructions. Briefly, 1×10^4 cells were seeded in Seahorse XF microplates. One hour before analysis, the culture medium was replaced with Seahorse XF assay media (Agilent Technologies, 103575-100), and plates were incubated at 37°C in a CO_2-free incubator. The mitochondrial stress test was conducted under basal conditions and following sequential injections of 1.5 μM oligomycin, 0.5 μM FCCP, and 0.5 μM rotenone/antimycin A. The Real-Time ATP Rate Assay was performed under basal conditions and after sequential injections of 1.5 μM oligomycin, and 0.5 μM rotenone/antimycin A.

Mitochondrial ROS measurement

Mitochondrial ROS production was assessed using the red MitoSox Superoxide indicator (Thermo Fisher Scientific, M36008), which detects ROS content specifically targeted to mitochondria. MitoSox Red reagent was dissolved in vehicle to make 5mM stock solution, and 5 μM working solution was prepared in HBSS. Vehicle or NAT-treated immortalized AT2 cells were incubated with 1 mL MitoSox working solution for 20 min and washed 3 times with warm PBS. Fluorescence signals were determined by flow cytometry. To validate the MitoSOox staining results, cells were treated with DMSO as a negative control and 50 μM CCCP as a positive control.

Cell growth rate

Cell growth rate of NAT treatment was measured by IncuCyte ZOOM Live Cell Analysis System (Essen BioScience) by following the instruction from the manufacturer.

NAD/NADH measurement

Total NAD and NADH were measured by NAD/NADH Quantification Kit (Sigma, Catalog MAK037) following manufacturer's instruction. 2×10^5 immortalized AT2 cells were washed with cold PBS, resuspended in 400 μl of NAD/NADH extraction buffer by freeze/thawing for 2 cycles of 20 minutes on dry ice followed by 10 minutes at room temperature. The extracted supernatant was split in half for total NAD+ and NADH after centrifugation at 13,000 × g. One-half sample was heated to 60 °C for 30 min to detect NADH. For total NAD measurement, 50 μl of sample was transferred into 96-well plate, and added with 100 μl of master reaction mix (NAD cycling buffer and enzyme mix from the kit), incubated for 5 min at room temperature to convert NAD to NADH. 10 μl of developer reagent was added to each well, and incubated at room temperature for 1 h. Absorbance at 450 nm was determined using a microplate reader. and their ratios were calculated by the following equation: ratio = $(NAD_{total} – NADH)/ NADH$.

Single-cell western blotting

Single-cell western blot analysis for NAMPT was performed following the manufacturer's instructions (Milo Single Cell Western Blot System). Briefly, single-cell suspensions of healthy and IPF lung AT2 cells were loaded onto single-cell chips (Standard scWest Kit, K600-1, Proteinsimple) and processed using the Milo system. After buffer washes, the chips were incubated with anti-NAMPT antibody (Cell Signaling Technology, Catalog #61122, RRID: AB_2799602, 1:40 dilution) and anti–β-tubulin antibody (GenScript, clone 2G7D4, Catalog # A01717, RRID: AB_2622210, 1:40 dilution) for 2 hours, followed by incubation with fluorescence-labeled secondary antibodies for 1 hour at room temperature. The chips were then read, and data were analyzed using the Scout system. Relative protein expression is quantified by calculating the fluorescence intensity ratio of NAMPT to β-Tubulin for each cell.

References

1. Liang J, et al. Hyaluronan and TLR4 promote surfactant-protein-C-positive alveolar progenitor cell renewal and prevent severe pulmonary fibrosis in mice. *Nat Med.* 2016;22(11):1285-93.
2. Tran E, et al. Development of human alveolar epithelial cell models to study distal lung biology and disease. *iScience.* 2022;25(2):103780.
3. Liang J, et al. A macrophage subpopulation recruited by CC chemokine ligand-2 clears apoptotic cells in noninfectious lung injury. *Am J Physiol Lung Cell Mol Physiol.* 2012;302(9):L933-40.
4. Wang L, et al. Optimization of NAMPT activators to achieve in vivo neuroprotective efficacy. *Eur J Med Chem.* 2022;236:114260.
5. Liang J, et al. Mitogen-activated Protein Kinase-activated Protein Kinase 2 Inhibition Attenuates Fibroblast Invasion and Severe Lung Fibrosis. *Am J Respir Cell Mol Biol.* 2019;60(1):41-8.
6. Liu X, et al. HER2 drives lung fibrosis by activating a metastatic cancer signature in invasive lung fibroblasts. *J Exp Med.* 2022;219(10).
7. Konishi S, et al. Defined conditions for long-term expansion of murine and human alveolar epithelial stem cells in three-dimensional cultures. *STAR Protoc.* 2022;3(2):101447.

Figure S1

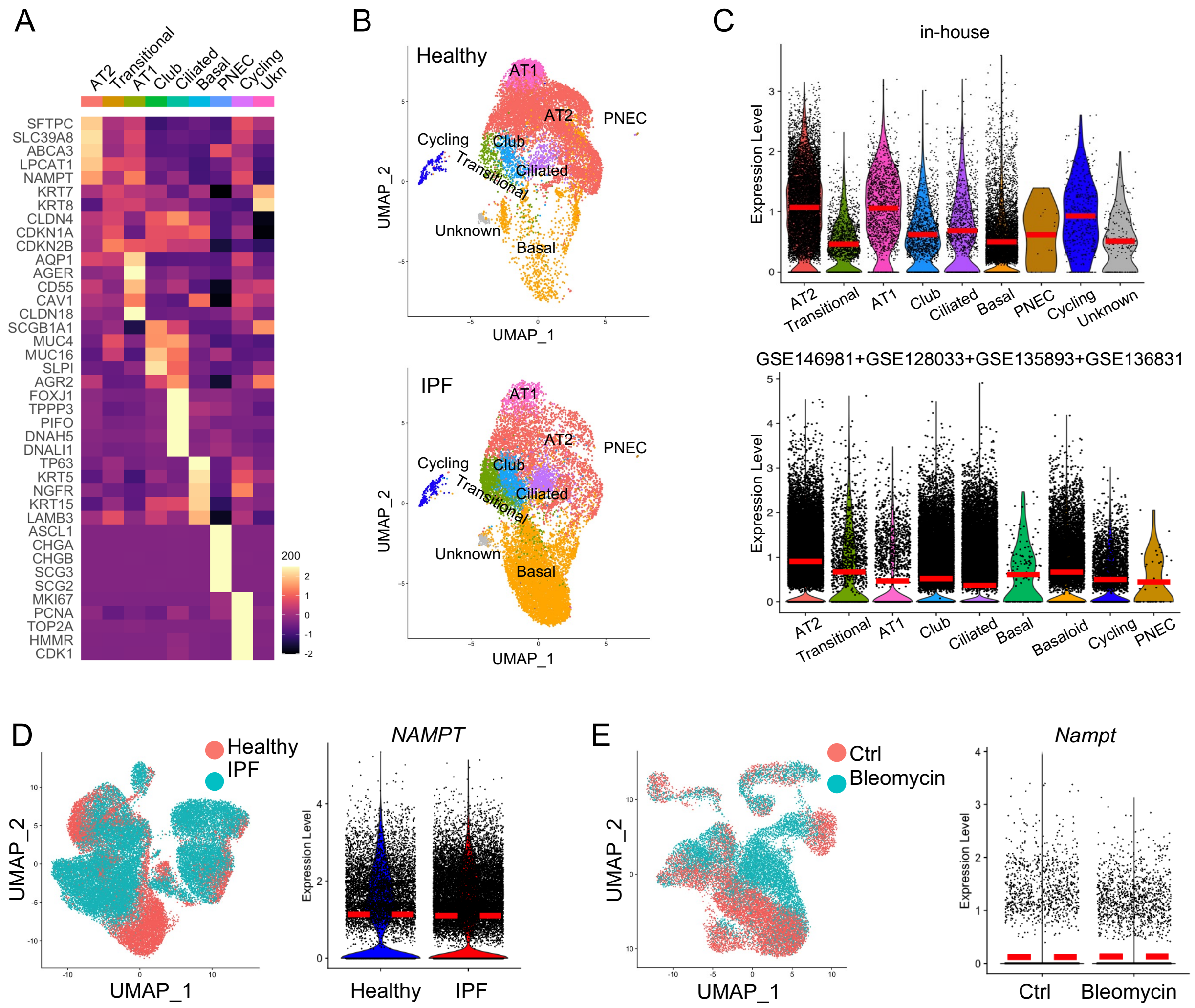

Figure S1. **NAMPT expression in epithelial cells and fibroblasts.**
(**A**) Heatmap showed the expression of canonical marker genes used for epithelial cell type annotations.
(**B**) Split UMAP visualization of epithelial cell types in in the in-house scRNA-seq dataset of healthy (top) and IPF (bottom) lungs.
(**C**) Violin plots showed *NAMPT* expression levels in lung epithelial cell types from in-house and integrated published scRNA-seq datasets.
(**D** and **E**) UMAP visualization of fibroblast populations and *NAMPT* expression in integrated datasets from 7 scRNA-seq datasets on healthy and IPF human lungs (**D**) (GSE136831, GSE157376, GSE128169, GSE135893, GSE128033, GSE122960, and GSE132771) and 5 scRNA-seq datasets on bleomycin-induced fibrotic mouse lungs and controls (**E**) (GSE111664, GSE129605, GSE131800, GSE104154, and GSE132771).

Figure S2

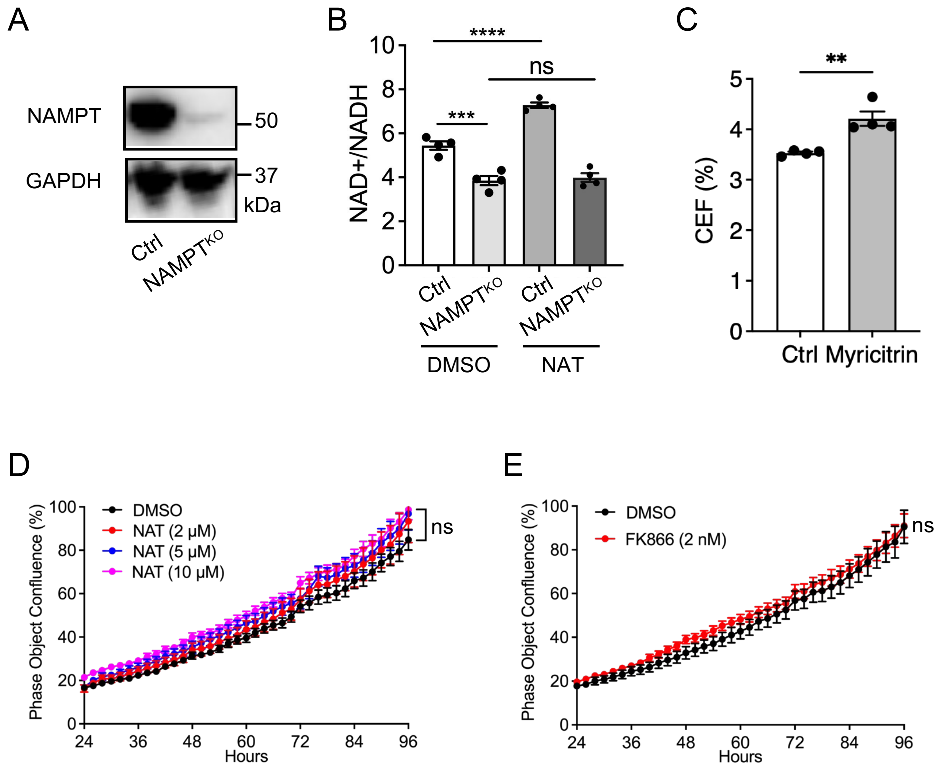

Figure S2. **NAT promotes NAD^+ biosynthesis in AT2 cells specifically through NAMPT.**
(**A**) Western blot analysis of NAMPT expression in $NAMPT^{KO}$ and control immortalized healthy AT2 cells. GAPDH served as loading control.
(**B**) NAD^+/NADH ration in $NAMPT^{KO}$ and control immortalized healthy AT2 cells with or without NAT treatment (n = 4 per group, ns, no significant, ***p < 0.001, ****p < 0.0001 by ANOVA).
(**C**) Colony forming efficiency (CFE) in 3D-organoid cultures of AT2 cells from healthy lungs treated with Myricitrin (25 μg/mL). (n = 4 per group, **p < 0.01 by unpaired two-tailed t test).
(**D** and **E**) Cell growth rate of immortalized healthy AT2 cells treated with NAT or FK866 at indicated concentrations (n = 6 per group, ns, no significant by unpaired two-tailed t tests or ANOVA).
Data are shown as the mean ± SEM.

Figure S3

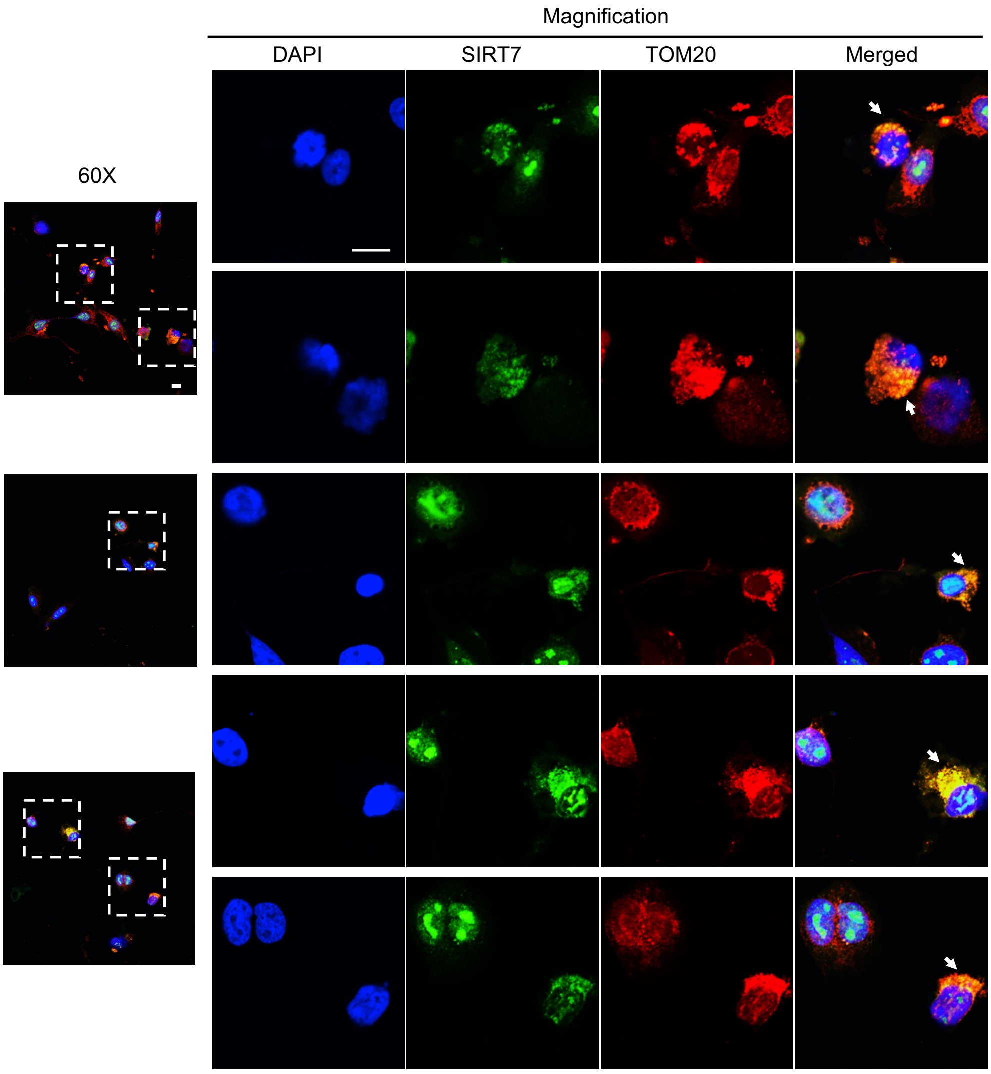

Figure S3. **Immunofluorescence co-staining of SIRT7 and TOM20 in human AT2 cells.**
Immunofluorescence co-staining of SIRT7 and TOM20 in immortalized healthy AT2 cells. Arrows indicated the co-staining of SIRT7 and TOM20 in boxed cells. Boxed areas are shown at higher magnification. Scale bar, 100 μm.
All experiments were repeated at least three times.

Figure S4

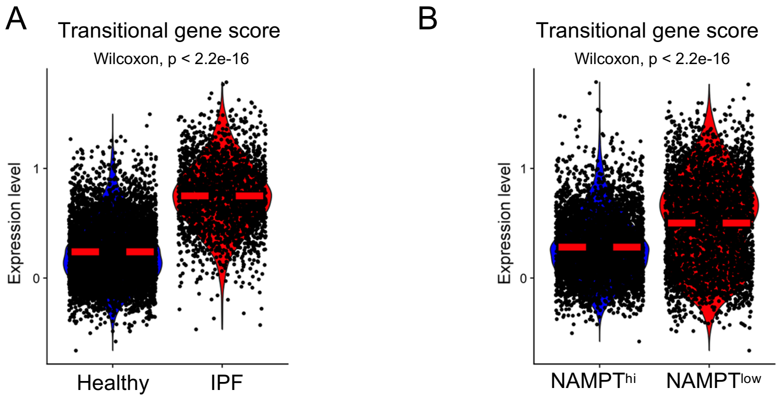

Figure S4. **Transitional signatures in AT2 cells.**
Transitional signatures in (**A**) healthy vs IPF and (**B**) NAMPT^{hi} vs NAMPT^{low} AT2 cells by the average expression of transitional cell marker genes: *KRT7, KRT8, KRT17, KRT18, KRT19, CLDN4, ITGB6, CDKN1A, CDKN2A, CDKN2B*.

Figure S5

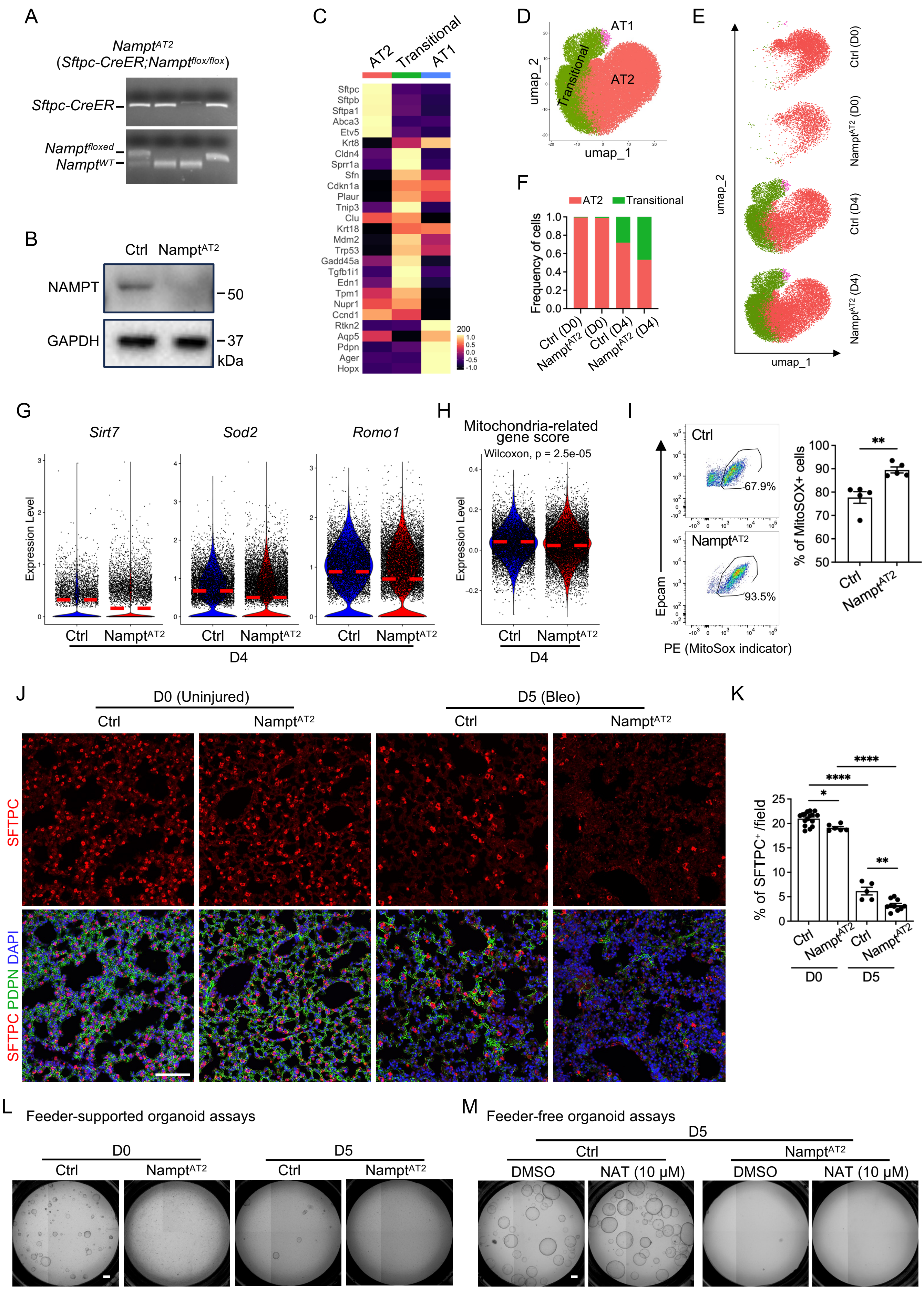
A
NamptAT2
(Sftpc-CreER;Namptflox/flox)
Sftpc-CreER
Namptfloxed
NamptWT
B
Ctrl
NamptAT2
NAMPT
GAPDH
50
37
kDa
C
AT2
Transitional
AT1
Sftpc
Sftpb
Sftpa1
Abca3
Etv5
Krt8
Cldn4
Sprr1a
Sfn
Cdkn1a
Plaur
Tnip3
Clu
Krt18
Mdm2
Trp53
Gadd45a
Tgfb1i1
Edn1
Tpm1
Nupr1
Ccnd1
Rtkn2
Aqp5
Pdpn
Ager
Hopx
D
AT1
Transitional
AT2
umap_1
umap_2
E
Ctrl (D0)
NamptAT2 (D0)
Ctrl (D4)
NamptAT2 (D4)
umap_1
umap_2
F
AT2
Transitional
Frequency of cells
Ctrl (D0)
NamptAT2 (D0)
Ctrl (D4)
NamptAT2 (D4)
G
Sirt7
Sod2
Romo1
Expression Level
Ctrl
NamptAT2
D4
H
Mitochondria-related gene score
Wilcoxon, p = 2.5e-05
Expression Level
Ctrl
NamptAT2
D4
I
Ctrl
67.9%
NamptAT2
93.5%
Epcam
PE (MitoSox indicator)
% of MitoSOX+ cells
**
J
D0 (Uninjured)
D5 (Bleo)
Ctrl
NamptAT2
SFTPC
SFTPC PDPN DAPI
K
% of SFTPC+ /field
*
****
****
**
Ctrl
NamptAT2
D0
D5
L
Feeder-supported organoid assays
D0
D5
Ctrl
NamptAT2
M
Feeder-free organoid assays
D5
Ctrl
NamptAT2
DMSO
NAT (10 μM)

Figure S5. **AT2-specific Nampt deletion leads to elevated oxidative stress and transitional cell accumulation after injury.**
(**A**) Representative result showed the genotyping of *Sftpc-CreER*; *Nampt$^{flox/flox}$* (*Nampt^{AT2}*) mouse.
(**B**) Western blot analysis of NAMPT expression in Nampt^{AT2} and control AT2 cells. GAPDH served as loading control.
(**C**) Heatmap showed the expression of canonical marker genes used for annotations of alveolar epithelial cell subtypes in the scRNA-seq.
(**D** and **E**) Integrative and split UMAPs showed the alveolar epithelial cell subtypes in the scRNA-seq data of alveolar epithelial cells from lungs of Nampt^{AT2} and control mice before (D0) and 4 days (D4) after bleomycin injury.
(**F**) Frequency of cells in alveolar epithelial cell subtypes in the scRNA-seq data of each group.
(**G**) Violin plots showed expression levels of oxidative stress response genes (*Sod2* and *Romo1*) and *Sirt7* in injured AT2 cells (D4) from Nampt^{AT2} and control mouse lungs.
(**H**) Mitochondrial-related gene score in injured (D4) AT2 cells from control and Nampt^{AT2} mouse lungs.
(**I**) Flow cytometry analysis of mitochondrial superoxide levels in AT2 cells isolated from Nampt^{AT2} and control mice 5 days after bleomycin injury ($n = 3$ per group, $^{**}p < 0.01$ by unpaired two-tailed t test).
(**J**) Representative images of immunostaining for AT2 (SFTPC) and AT1 (PDPN) markers on the lung sections from control (D0 uninjury) and 5 days after bleomycin-injured (D5 Bleo) Nampt^{AT2} and control mice. Scale bar, 100 μm.
(**K**) Quantification of the percentages of SFTPC^{+} AT2 cells on each lung sections from D0 and D5 Bleo Nampt^{AT2} and control mice (n = 16, 6, 5, 10 per group, respectively. $^{*}p < 0.05$, $^{****}p < 0.0001$ by ANOVA).
(**L**) Representative images of MLg2908 feeder-supported 3D-organoid cultures of AT2 cells from Nampt^{AT2} and control mice uninjured (D0) or 5 days after bleomycin injury (D5). Scale bars, 300 μm.
(**M**) Representative images of feeder-free 3D-organoid cultures of AT2 cells from Nampt^{AT2} and control mice 5 days after bleomycin injury (D5). Scale bars, 300 μm.
Data are shown as the mean ± SEM. All experiments were repeated at least three times.

Figure S6

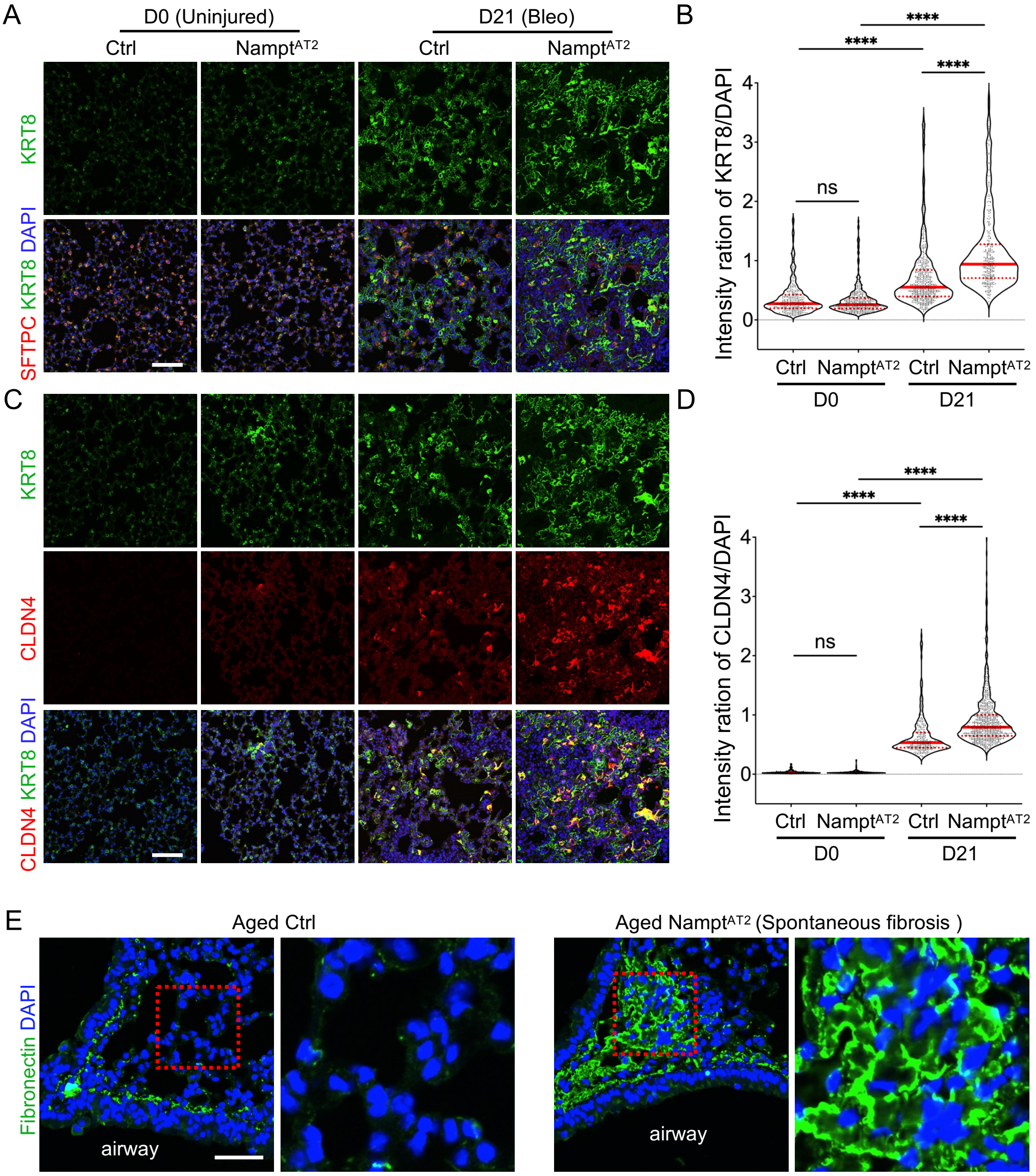

Figure S6. **AT2-specific Nampt deletion leads to increased transitional cell accumulation in bleomycin-induced fibrosis model.**
(**A** and **C**) Representative images showed the (**A**) co-immunostaining for AT2 (SFTPC) and transitional cell (KRT8) markers and (**B**) co-immunostaining for transitional cell markers (KRT8 and CLDN4) on lung sections from control (D0 uninjuried) and fibrosis (D21 Bleo) lungs from Nampt^{AT2} and control mice. Scale bars, 100 μm.
(**B** and **D**) Quantification of the intensity ratio of (**B**) KRT8 relative to DAPI per cell and (**D**) CLDN4 relative to DAPI per cell on lung sections from control and fibrosis lungs from Nampt^{AT2} and control mice (n > 300 cells per group, ****p < 0.0001 by ANOVA).
(**E**) Representative images showed the immunostaining for fibronectin on lung sections of Nampt^{AT2} at the age of 14 months showed excessive Fibronectin deposition in area of increased cellularity. Boxed areas are shown at higher magnification. Scale bars, 50 μm.
All experiments were repeated at least three times.

Figure S7

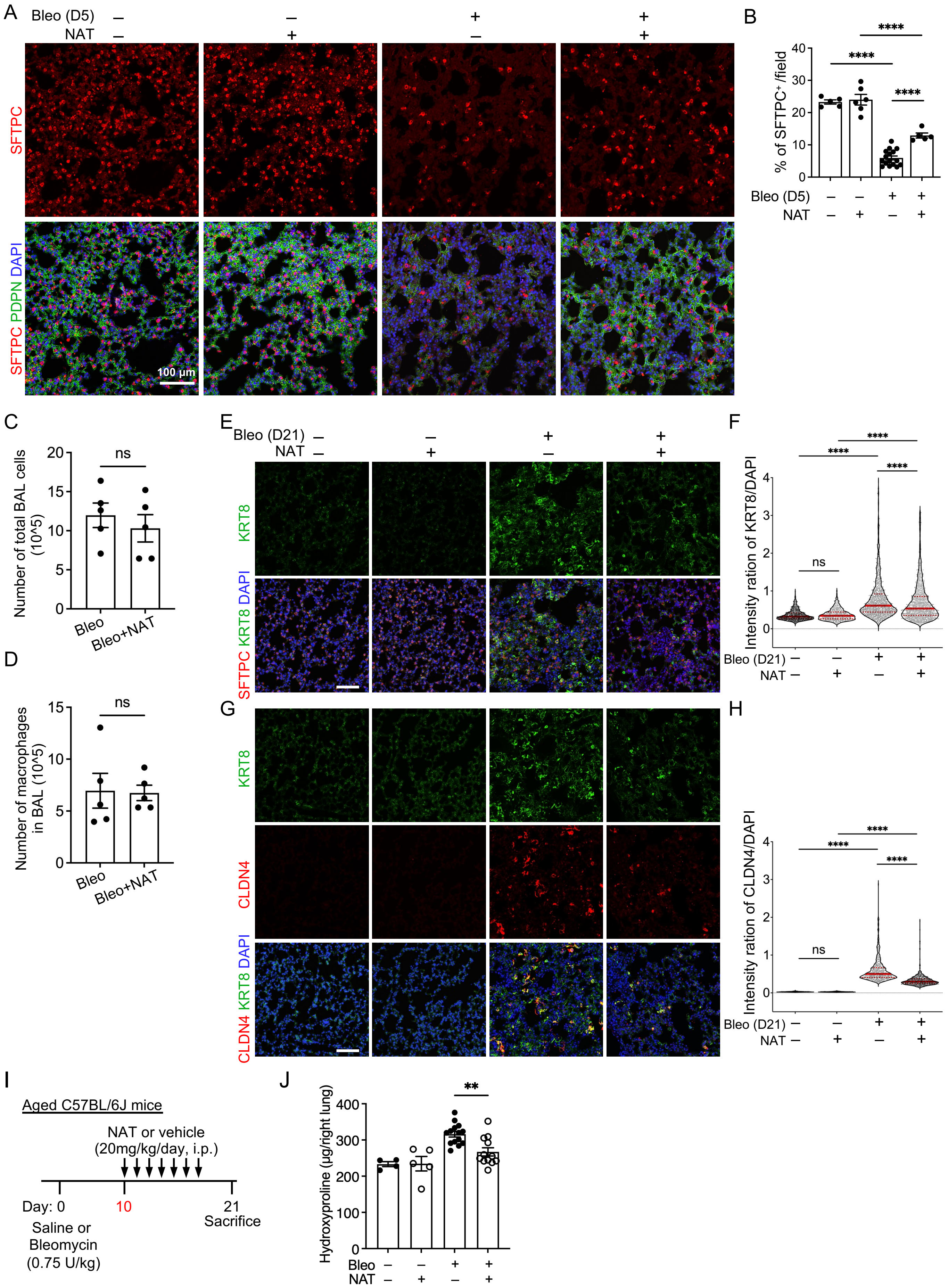
A
Bleo (D5)
NAT
SFTPC
SFTPC PDPN DAPI
100 µm
B
% of SFTPC⁺ /field
****
Bleo (D5)
NAT
C
Number of total BAL cells (10^5)
ns
Bleo
Bleo+NAT
D
Number of macrophages in BAL (10^5)
ns
Bleo
Bleo+NAT
E
Bleo (D21)
NAT
KRT8
SFTPC KRT8 DAPI
F
Intensity ration of KRT8/DAPI
ns
****
Bleo (D21)
NAT
G
KRT8
CLDN4
CLDN4 KRT8 DAPI
H
Intensity ration of CLDN4/DAPI
ns
****
Bleo (D21)
NAT
I
Aged C57BL/6J mice
NAT or vehicle
(20mg/kg/day, i.p.)
Day: 0
10
21
Sacrifice
Saline or
Bleomycin
(0.75 U/kg)
J
Hydroxyproline (µg/right lung)
**
Bleo
NAT

Figure S7. **NAMPT activation *in vivo* promotes AT2 regeneration and reduces transitional cell accumulation in fibrosis model.**
(**A**) Representative images of immunostaining for AT2 (SFTPC) and AT1 (PDPN) markers on the lung sections from uninjured and 5 days after bleomycin-injured (D5 Bleo) young C57BL/6J mice pre-treated with NAT or vehicle. Scale bar, 100 μm.

(**B**) Quantification of the percentages of $SFTPC^+$ AT2 cells on each lung sections from uninjured and bleomycin-injured (D5 Bleo) C57BL/6J mice with or without NAT treatment (n = 5, 6, 16, 5 per group, respectively. *p < 0.05, ****p < 0.0001 by ANOVA).

(**C**) Numbers of total BAL cells and (**D**) numbers of macrophages in BAL per lung of mice treated with NAT and bleomycin or with bleomycin alone for 5 days as indicated in figure 7A (n = 5 per group, ns, no significant by unpaired two-tailed t test).

(**E** and **G**) Representative images showed the (**E**) co-immunostaining for AT2 (SFTPC) and transitional cell (KRT8) markers and (**G**) co-immunostaining for transitional cell markers (KRT8 and CLDN4) on control (D0 uninjuried) and fibrosis (D21 Bleo) lung sections from young C57BL/6J mice pre-treated with NAT or vehicle. Scale bars, 100 μm.

(**F** and **H**) Quantification of the intensity ratio of (**F**) KRT8 relative to DAPI per cell and (**H**) CLDN4 relative to DAPI per cell on lung sections from young C57BL/6J mice pre-treated with NAT or vehicle (n > 300 cells per group, ****p < 0.0001 by ANOVA).

(**I**) Experiment layout: Aged C57BL/6J wild-type mice were administrated with 0.75 U/kg bleomycin and were treated with 20 mg/kg NAT at fibrotic stage (day 10-16), and the lungs were collected on day 21 after bleomycin treatment.

(**J**) Hydroxyproline in the lungs (n = 5-14 per group, **p < 0.01 by ANOVA) were used to assess collagen levels in mouse lungs.

Data are shown as the mean ± SEM. All experiments were repeated at least three times.

Supplemental Table 1. Gene lists used for the gene set scores

Mitochondria related genes	Transitional cell marker genes
CALM1	KRT7
CARM1	KRT8
CHD9	KRT17
CRTC2	KRT18
GLUD1	KRT19
GLUD2	CLDN4
HELZ2	SFN
MAPK14	ITGB6
MEF2D	CDKN1A
PPRC1	CDKN2A
PRKAA2	CDKN2B
PRKAB2	
PRKAG2	
SOD2	
TFAM	
TFB1M	
TFB2M	
TWNK	
PINK1	
COX5B	
MFN2	
