# Supplementary material for: Activation of the impaired NAMPT/SIRT7/SOD2 axis restores alveolar progenitor cell renewal in idiopathic pulmonary fibrosis
Source: J Clin Invest. 2026 May 7;136(12):e198031. doi: 10.1172/JCI198031 (PMC13262719; doi:10.1172/JCI198031)

Full unedited blot/gel for Figure 1F

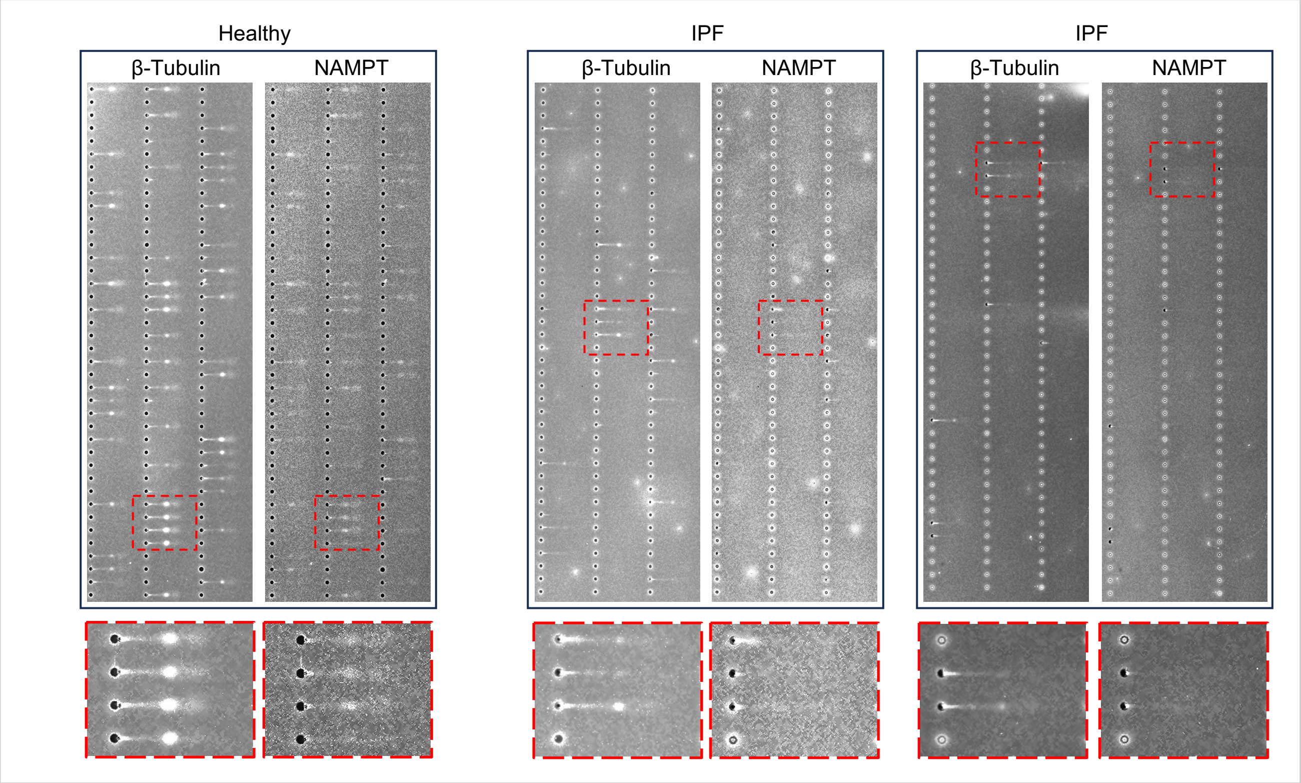

Full unedited blot/gel for Figure 1G

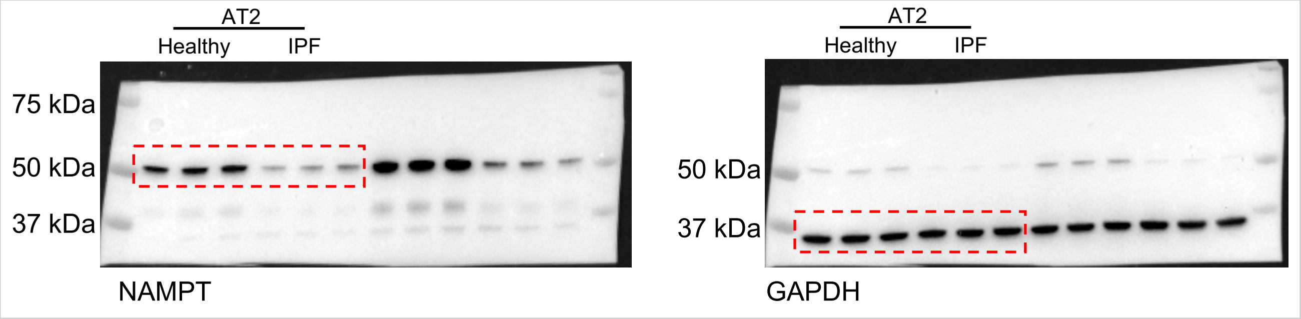

Full unedited blot/gel for Figure S2A

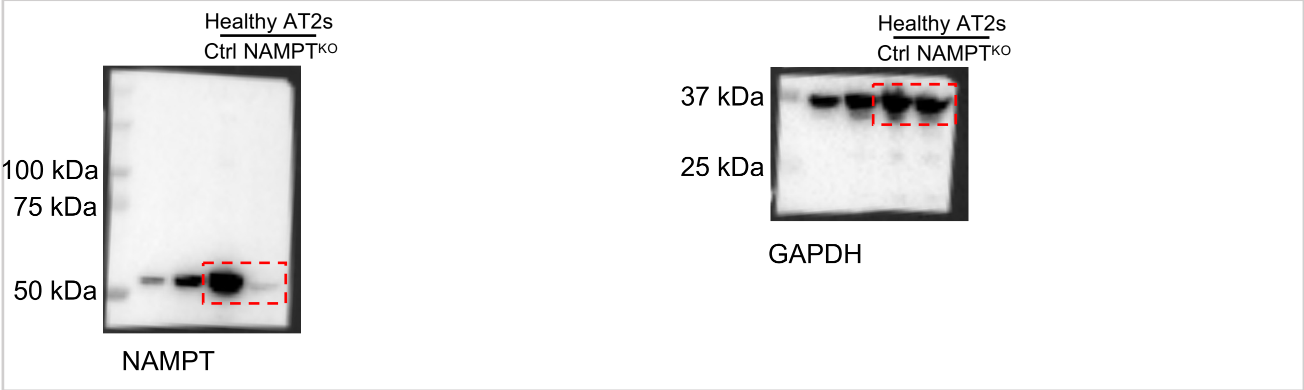

Full unedited blot/gel for Figure 4D

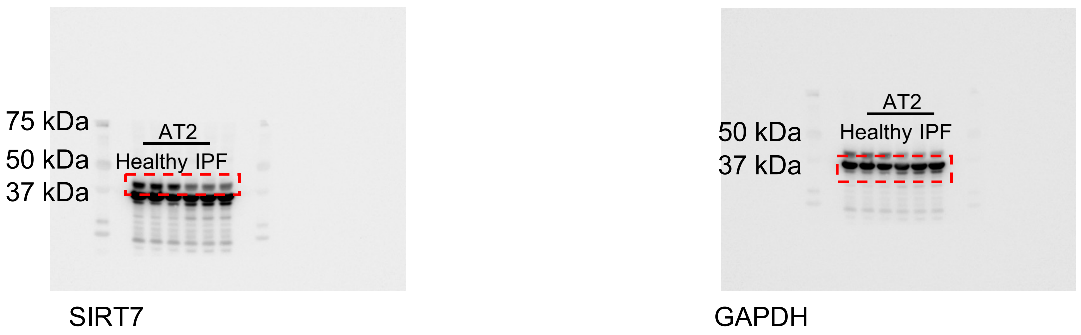

Full unedited blot/gel for Figure 4E

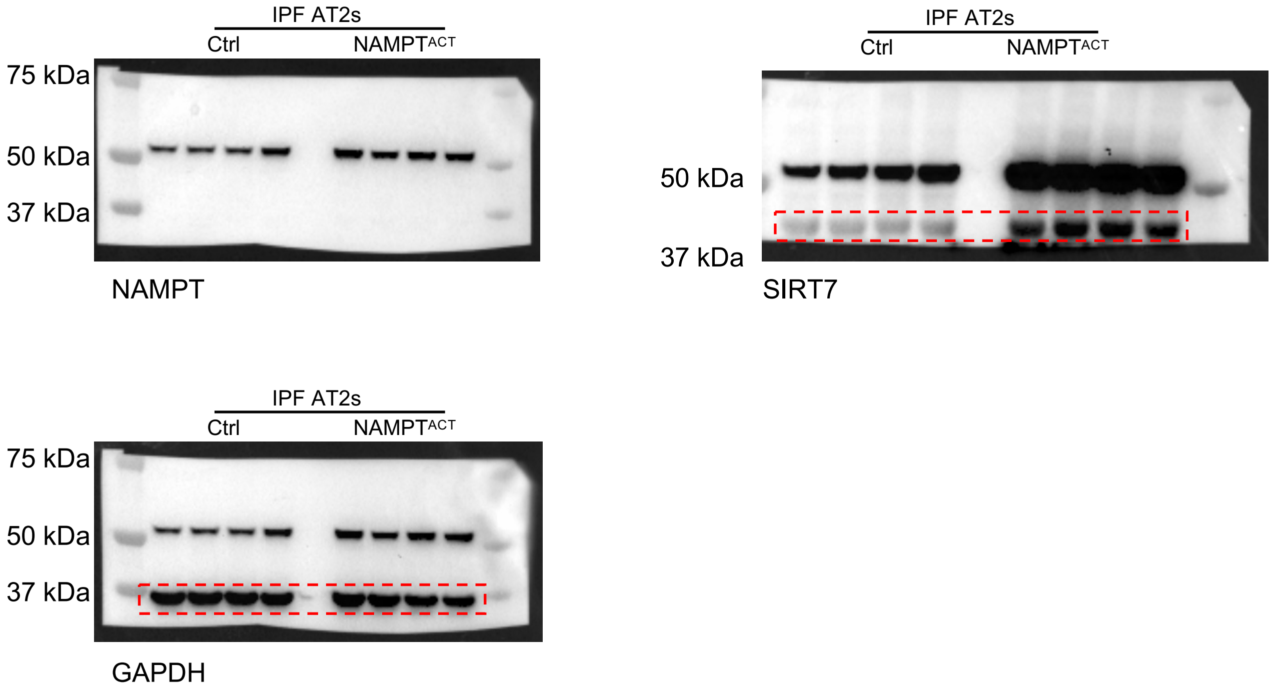

Full unedited blot/gel for Figure 4F

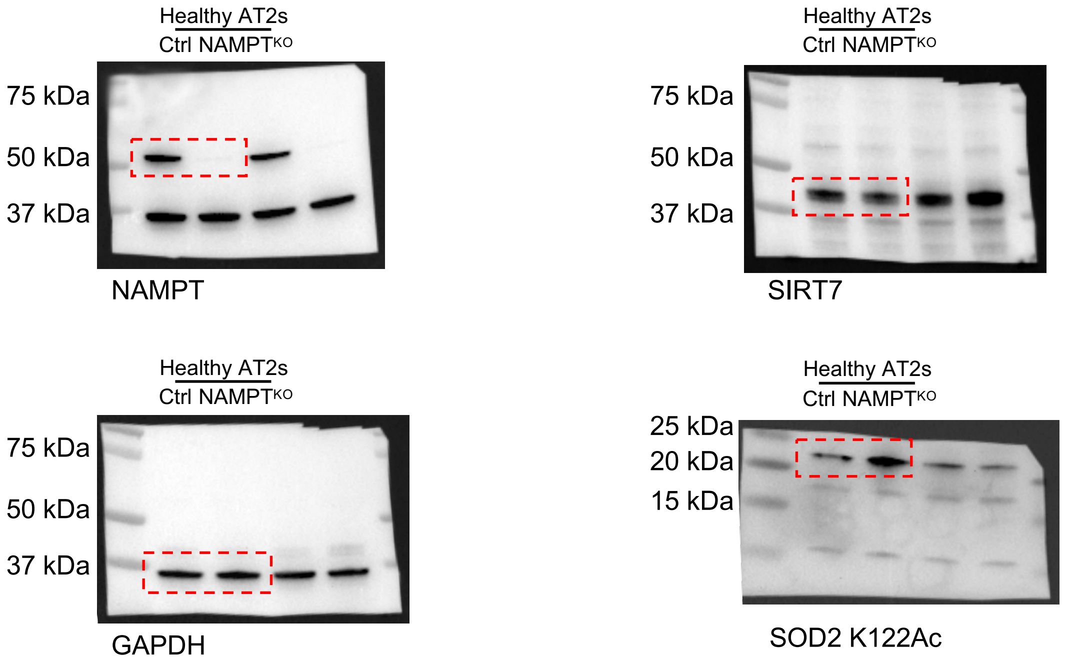

Full unedited blot/gel for Figure 4G

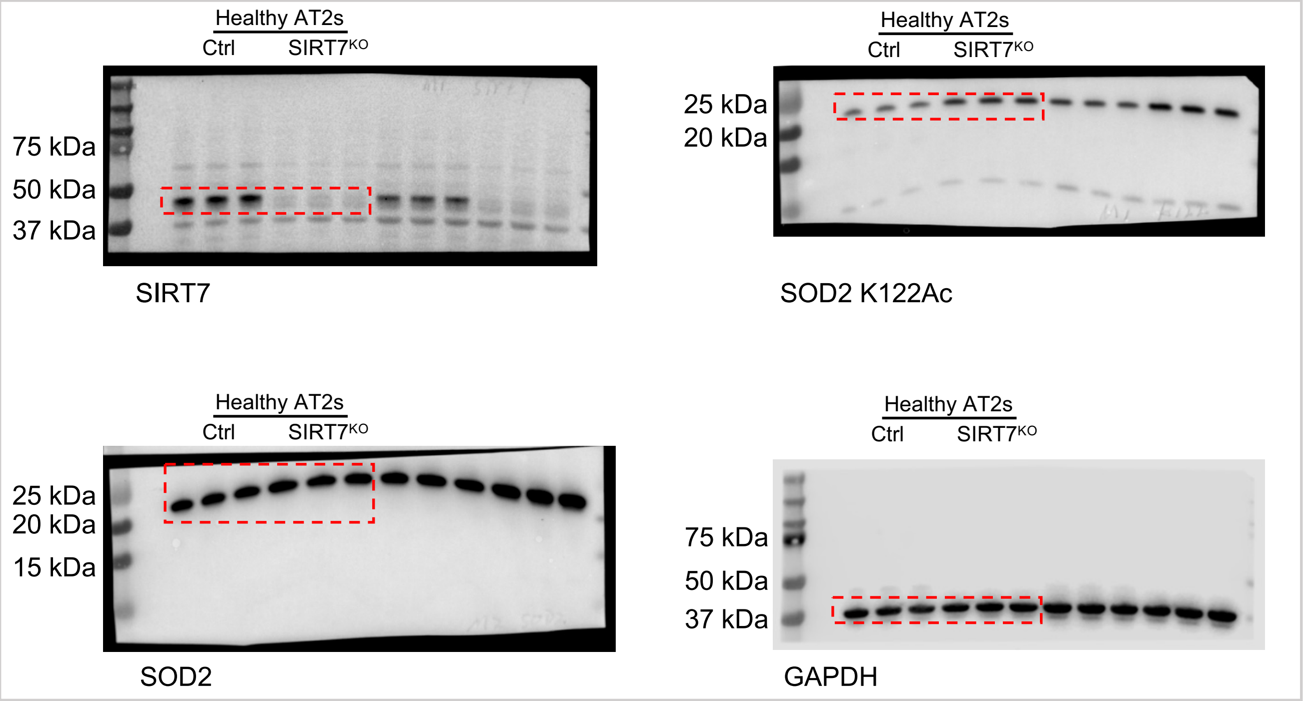

Full unedited blot/gel for Figure 4H

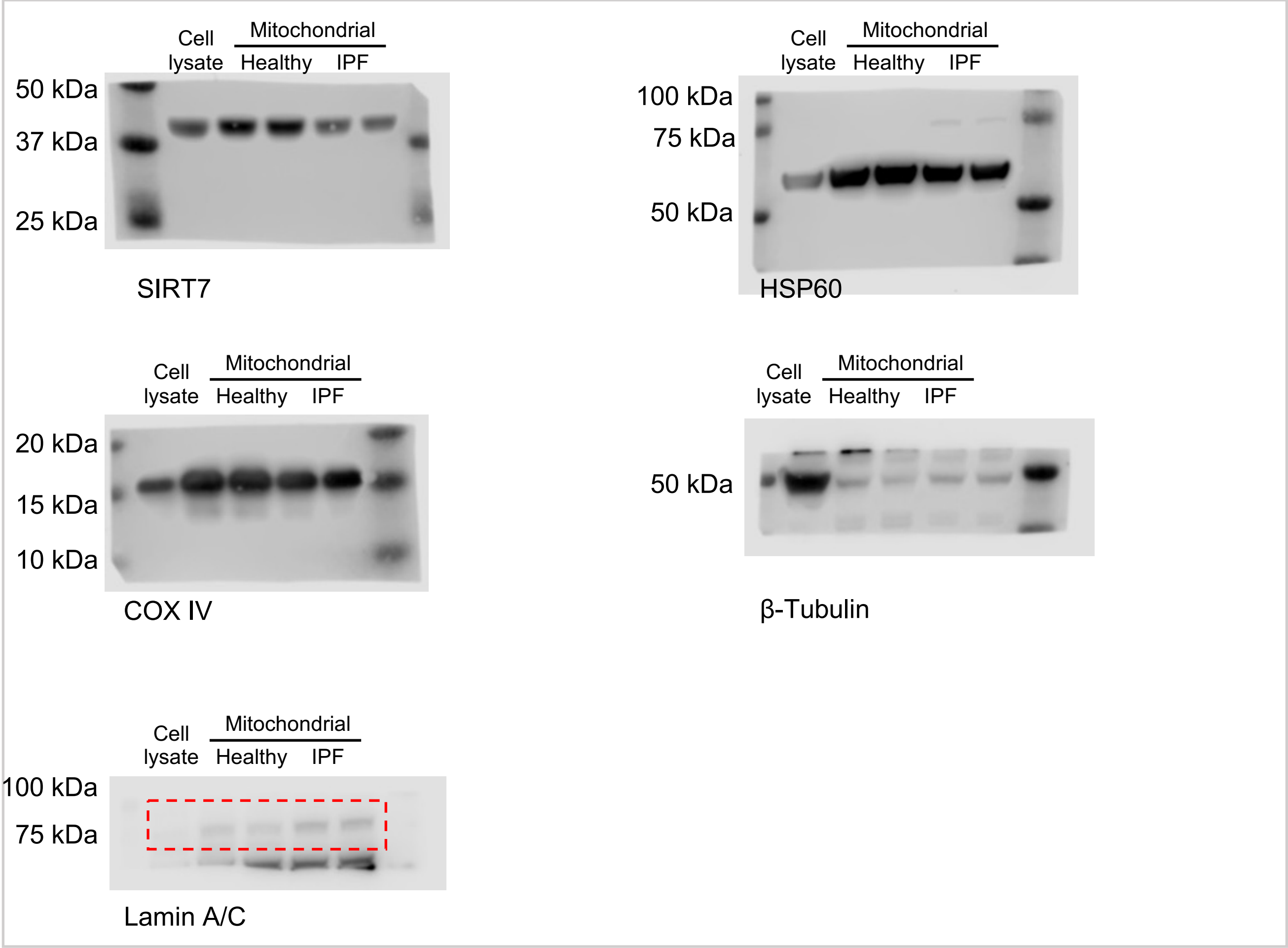

Full unedited blot/gel for Figure S5B

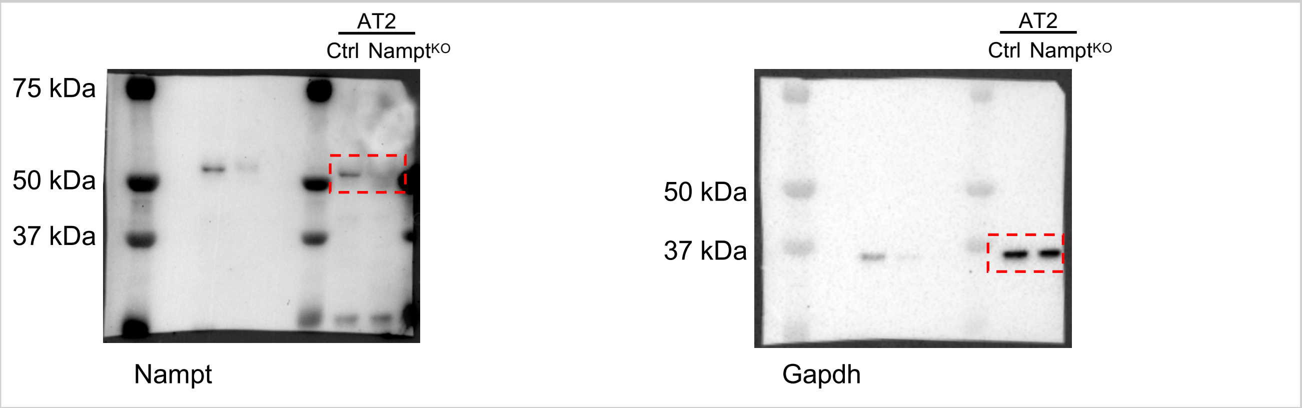

Full unedited blot/gel for Figure 8A

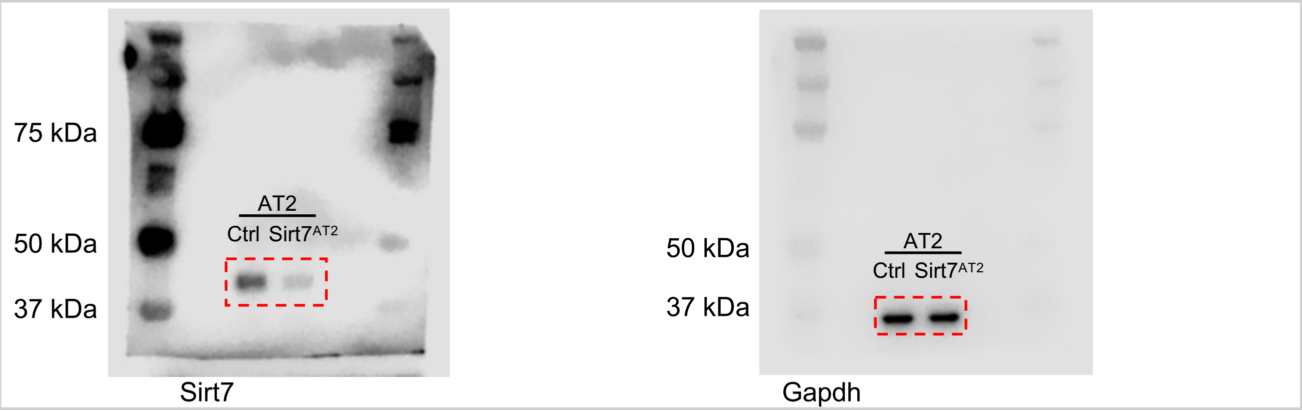

Supplement: Unedited blot and gel images [file jci-136-198031-s210.pdf]
